# Exploring HIV prevention preferences among key populations in Uganda: A qualitative study

**DOI:** 10.1371/journal.pone.0349414

**Published:** 2026-06-08

**Authors:** Maiya G. Block Ngaybe, Kawoya K. Joseph, Wadana Hamzazai, Carly J. Deal, Stephen Mugamba, Wambi M. Stephen, Richard Muhumuza, Betty Nakaggwa, Auwal Abubakar, Gabriela Valdez, Maia Ingram, John Ehiri, Grace Mirembe, Betty Mwesigwa, Hannah Kibuuka, Purnima Madhivanan

**Affiliations:** 1 Department of Health Promotion Sciences, Mel and Enid Zuckerman College of Public Health, University of Arizona, Tucson, Arizona‌‌, United States of America; 2 Makerere University Walter Reed Program, Kampala, Uganda; 3 Medical Research Council/Uganda Virus Research Institute and London School of Hygiene and Tropical Medicine Uganda Research Unit, Entebbe‌‌, Uganda; 4 Public Health Research Institute of India, Mysore, Karnataka, India; Medical Research Council, SOUTH AFRICA

## Abstract

Long-acting injectable pre-exposure prophylaxis (PrEP) has recently been approved for use, and dissemination, but has yet to be released in Uganda. To ensure uptake of this injectable option, and others like a future HIV vaccine, it is important to understand the preferences of the populations at the highest risk who will benefit the most. We present data on product preferences for HIV prevention practices and injectable HIV prevention among populations most at risk for HIV with a focus on injectable options such as long-acting PrEP or a future HIV Vaccine for primary prevention of HIV. From March 18–28, 2024, we conducted 20 semi-structured key informant interviews in English and Luganda among 10 experts in the field of HIV prevention, and 10 peer leaders of key or priority populations in Uganda. Participants were purposively selected to represent various groups, genders, occupations and locations to get diverse perspectives. Participants were included if they were determined to be at risk of contracting HIV and were above the age of 18. Both groups of participants were asked similar questions, with experts focusing on their experience with key populations, populations who are particularly vulnerable to HIV acquisition due to a combination of behavioral, biological, and social factors. Debriefs were held after each interview to monitor emerging themes and assess data saturation. The most common prevention practices mentioned by participants were condoms and PrEP. Participants mentioned barriers to prevention practices including duration (i.e., dosing length), accessibility issues such as transportation, location issues (i.e., distance), and stigma. The most important characteristics for participants when considering the uptake of a new injectable prevention product included efficacy, cost and side effects. Experts tended to believe that efficacy levels should be higher than peer leaders with lived experience. Participants who were peer leaders recognized that some had a fear of needles, though they also expressed motivation to receive injections due to the perception that they may work better than other modes of administration. According to experts and peer leader participants in this study, key populations in Uganda prefer high efficacy, lower side effects and confidentiality in their services. Participants emphasized the need for comprehensive and accessible information about specific HIV prevention methods to improve the uptake of these products. Tailored messaging and choice can accommodate the heterogeneity of preferences to best ensure HIV prevention in Uganda.

## Introduction

Human immunodeficiency virus (HIV) affects approximately 39.9 million people worldwide with an incidence rate of 1.3 million individuals per year [1,2]. Sub-Saharan Africa bears a disproportionate burden of the global HIV epidemic, with Uganda ranking among the top five countries with the highest prevalence of people living with HIV [3]. The national prevalence hovers around 5.4%, with a higher concentration of cases in urban areas and among key populations, e.g., people who sell or exchange sex, people who inject drugs (PWID), men who have sex with men (MSM), and transgender individuals; these key populations are particularly vulnerable to HIV acquisition due to a combination of behavioral, biological, and social factors [4]. Key population groups often face structural barriers, including criminalization, stigma, and limited access to healthcare services, which further exacerbates their risk [5]. Among the general population, young adults, particularly women and adolescent girls, are at an elevated risk of acquiring HIV [6]. In sub-Saharan Africa [7], young women are twice as likely as young men to be living with HIV, a disparity driven by gender inequalities, limited access to sexual and reproductive health services, and higher levels of gender-based violence [6]. Addressing these vulnerabilities requires not only medical solutions but also comprehensive strategies that reduce stigma and discrimination while increasing access to care.

Despite advancements in treatment, HIV remains a major global public health challenge. HIV is a retrovirus that compromises the immune system by targeting CD4 + T cells, weakening the body’s ability to fight infections and increasing vulnerability to opportunistic diseases. This virus is particularly challenging to eliminate due to its ability to integrate into the host’s DNA, forming latent reservoirs that are not easily targeted by antiretroviral therapies (ART) [8]. Additionally, HIV’s rapid mutation rate complicates the development of a universal cure or vaccine [8]. The complexity of HIV’s structure, its mutability, and the challenge of targeting the virus in latent reservoirs in particular have hindered progress in vaccine development [8].

Over the past few decades, considerable progress has been made in the development of HIV prevention technologies. Current preventative measures include oral and injected pre-exposure prophylaxis (PrEP), the vaginal dapivirene ring, and condom use, yet these methods face challenges related to adherence and consistent use [9,10]. For populations with limited autonomy over their health decisions or those in stigmatized environments, the logistical and social challenges of maintaining daily PrEP regimes can be significant. The advent of long-acting injectable PrEP represents a promising advancement, offering sustained protection with a reduced burden on daily adherence [11,12].

In Uganda, HIV prevention for people at substantial risk commonly includes condoms, HIV testing, post-exposure prophylaxis (PEP), and daily oral PrEP delivered through public-sector and partner-supported services [13,14]. However, access and persistence remain uneven due to stigma, pill burden, transport and refill barriers, and variability in availability across health centers [15,16]. These contextual constraints shape how new long-acting injectable options may be perceived and used. In particular, Uganda’s HIV prevention strategy has expanded significantly in recent years with scale-up of PrEP delivery through public ART clinics, targeting key populations such as adolescent girls, sex workers, and transgender women [17,18]. Despite these gains, in this setting, daily oral prevention can be difficult to sustain due to pill burden, stigma tied to PrEP packaging resembling ART, and refill barriers like transportation, particularly for mobile or highly stigmatized groups [13,19]. Long-acting injectables may address these constraints by reducing dosing frequency and increasing discretion.

Prior preference research in Uganda and comparable settings suggests that attributes such as effectiveness, dosing frequency, discretion, and affordability shape acceptability of PrEP modalities and future biomedical prevention products [20]. For example, mixed-method work among adolescent girls and young women in Kampala reported interest across novel modalities, including vaccines and injectables, and a Uganda-based discrete choice experiment (DCE) among fisherfolk identified effectiveness and discretion as influential attributes [20,21]. However, there remains limited qualitative evidence triangulating expert and key-population perspectives to inform locally grounded attribute and level selection for injectable prevention options prior to rollout [22].

Longer-term preventative options under consideration include not only injectable PrEP but also implants and vaccines [11]. There is currently no approved HIV vaccine in Uganda or anywhere else; recent results from the PrEPVacc trial, which included a Masaka, Uganda site, found that the tested vaccine regimens did not reduce HIV infections, so routine vaccine access in Uganda is not expected in the near term [11,23]. By contrast, injectable PrEP is closer to access, with cabotegravir already recommended by WHO and Uganda engaged in CAB-LA adoption and implementation planning, although access appears to be phased rather than universal [11,24]. The recent approval of long-acting injectable PrEP marks a pivotal moment in HIV prevention. This new method provides discreet, sustained protection for high-risk populations, offering a pathway to overcome some of the social challenges associated with existing options [7]. Since the form of delivery of a vaccine and injectable PrEP are so similar, it is important to investigate differences in perceived benefits and costs of each injectable method to better understand future possible injectable product uptake.

In summary, investigating how key populations, particularly those in Uganda, value features of long-acting prevention strategies, such as injectable PrEP, will be crucial in shaping the future landscape of HIV prevention [7]. This information is essential to inform healthcare providers, policymakers, and intervention designers on how best to facilitate access and adoption of these novel technologies, ensuring that they meet the needs of those most vulnerable to HIV acquisition. This study examined factors that are most preferable to at-risk populations and therefore affect their decisions to accept a preventative HIV vaccine in comparison to other preventative methods with the primary objective to inform the implementation of a DCE in Uganda.

## Materials and methods

### Site

The Makerere University Walter Reed Project (MUWRP), the site where this protocol was implemented, is a biomedical research organization located in Kampala, Uganda. MUWRP has a long history of vaccine and therapeutic research in infectious diseases including HIV, Ebola, Marburg and tropical neglected diseases, such as schistosomiasis. MUWRP is also supported by the U.S. President’s Emergency Plan for AIDS Relief (PEPFAR) for comprehensive HIV prevention and treatment in four districts in central Uganda. Interviews took place at MUWRP HIV Clinics and over ten partnering sites that served key populations. These sites were either organizations that worked with key populations or HIV clinics and were utilized to make it more convenient for participants, to reduce costs of transportation and to increase confidentiality of interview engagements. Interviews took place primarily in Kampala, the capital city of Uganda with approximately 1.5 million (SD:1.4,1.6 mill) people living with HIV (PLWH) as of 2023 [4].

### Population‌‌

The study population included experts in the field of HIV prevention, and People at Substantial Risk for HIV Acquisition (PSRHA) in Uganda. HIV experts were recruited based on their experience working with at-risk populations, distributing or prescribing HIV prevention products, or consulting with at-risk populations regarding their HIV prevention choices with a specific emphasis on the following categories: Nurses, peers/community workers, prevention program managers, policy makers. Additionally, demographic representation such as gender, location and age was considered to account for demographic diversity in the sample. Kampala, Kayunga and Mukono were selected as the three sites for recruitment to represent three different major urban and peri-urban sites in Uganda. These were additionally sites where MUWRP had sites. MUWPR additionally has an existing community advisory board at Kayunga who was engaged as a part of this study.

PSRHA were defined in this study based on an adapted shortened criteria version of the Pre-Exposure Prophylaxis (PrEP) Screening for (High) Substantial Risk and Eligibility form used by the Ugandan Ministry of Health (HIMS ACP 028, 2019). These criteria included: being sexually active AND reporting vaginal or anal intercourse without condoms with more than one partner OR having a sex partner with one or more HIV risk(s), OR having a history of a sexually transmitted (STI), OR having a history of use of post-exposure prophylaxis (PEP), OR if an injection drug user, reports a history of sharing injection materials/equipment, OR has a sexual partner that is HIV positive and has not been on effective HIV treatment (see S1 File for more detail on inclusion criteria). Other criteria from the PrEP Screening Form were not included because they asked questions that were considered too sensitive at the time of the study commencement due to the country’s enactment of the Anti-Homosexuality Act [25] and may have endangered participants. For example, at initial stages of the study there was a policy which indicated that organizations were mandated to report LGBT populations to the Ugandan government, although the policy changed during the study. Participants were excluded from the key informant interviews if they were under the age of 18.

A sample size of 20 participants was determined to be sufficient to reach data saturation a priori based on previous literature and due to limited funding [26]. We therefore recruited ten experts in the field of HIV prevention and ten PSRHA. Our research team debriefed after each interview to discuss the degree of saturation of the data reached to determine if more participants were required past the original 20 planned. The team mutually decided that there was no need for additional recruitment after the last participant completed the interview as no new major themes were emerging.

### Community engagement and recruitment

We consulted with the MUWRP community advisory board (CAB) and asked for their advice before beginning community engagement. They provided feedback about best methods for recruitment and maintaining confidentiality and safety of participants. At later stages, they were presented results for member checking and aided in sharing results out to their respective communities. Participants in this study were purposively sampled with the help of MUWRP Community Engagement team to gather data from diverse sources using the MUWRP existing networks from previous MUWRP clinical research studies or PEPFAR activities. Participants were called by the MUWRP community engagement team using their existing community networks and understanding of the community using respondent-driven sampling techniques. Participants were consulted to determine the best meeting time and place for safety and confidentiality.

Research assistants conducted recruitment either via phone calls or face-to-face. Participants for the expert interviews were workers at HIV prevention programs in Kampala, Mukono, and Kayunga, HIV researchers, leaders of network organizations of the key populations, and researchers currently working on the HIV clinical trials. Experts and PSRHA were screened to ensure eligibility before the interview commenced. All participants who were engaged were interviewed, and there were no drop-out participants.

Due to the sensitive nature of the questions, and concerns for the safety of participants, some protective measures were put into place. For instance, if participants were unable to complete the interview at that time, they had the option to complete the process either at MUWRP or in the field at a later appointment time. Participants were also gender matched with the research assistant for the interviews as often as possible. Interviews were held in a private location where the conversation could not be overheard by others, for example a private room in a clinic or an office with a door that could be closed for privacy. Interviewers were trained to identify if the participant was uncomfortable. If they perceived that a participant was uncomfortable at any time, they reminded the participant that they could exit the study at any time.

Participants were also given the opportunity to ask any questions, or request that the research team delete their data if they finally decided that they did not want to be a part of the study in the end. Data collected during this study was stored with utmost care and confidentiality in double locked rooms if in person or encrypted secure shared drives with limited accessibility.

### Data collection

Interviews took place from March 18–28, 2024. The timeframe was limited due to limited funding and a strict timeframe for the project to conduct the subsequent discrete choice experiment. Before data collection took place, all study team members who took part in data collection activities took part in training on qualitative research methods, probing techniques, cultural context, field notes and transcription best practices. All consent, screening and data collection procedures took place in person. After consenting participants, research assistants asked them to provide some demographic information (S2 File). The demographic and eligibility questionnaires were administered on a tablet or on paper, if the tablet was not available or working. The research assistants then proceeded to data collection. There were two trained research assistants at each session, alternating roles for each interview. One research assistant acted as the interviewer, asking questions from participants, and the other acted as a notetaker, responsible for taking field notes in a structured format, on body language and tone, as well as audio recording interviews, keeping time, and suggesting probes to the interviewer as needed. The notes for each interview were taken in a matrix format structured according to similar questions in the guide to facilitate the rapid analysis process, described in more detail below.

The semi-structured key informant guides were created in English based on a previous similar DCE key informant interview guide [27]. The guide was translated by vetted contracted translators into Luganda, the most spoken local language in Kampala, Kayunga and Mukono (S1 File). The focus of the interview was to identify preferences of Ugandan key and priority populations regarding a future HIV vaccine or injectable preventative medication and to derive important factors that could influence their decision in receiving this medication, to be used to design a discrete choice experiment [28].

Topics discussed in expert and PSRHA interviews included: HIV vaccine in general (e.g., “what have you heard about the vaccine?”), preferred injectable characteristics (e.g., side effects, duration, effectiveness, frequency of administration, cost, dissemination points, person who dispenses), and other relevant health promotion and preference questions (e.g., feasibility of recruitment, messaging, moral foundations). The expert interview guide additionally focused on the preferences of the PSRHA with whom they work in their workplace, rather than on their personal preferences. The guide for expert participants had a few additional questions regarding approval and dissemination processes for a future HIV vaccine. The guide was pilot tested ahead of actual data collection within the study team.

Interviews lasted between 60 and 90 minutes. Interview duration varied primarily due to participant time constraints and differences in topic familiarity. Interviewers used a standard set of probes to ensure key domains were covered while allowing depth where participants had more experience. After each interview, the research assistant thanked the participant and provided 50,000 UGX (approximately 13.64 USD) as compensation for their time and transportation costs. Following the interview, the notetaker and interviewer debriefed together, or with a senior research team member to take note of any thoughts which emerged as part of the data collection process to inform the preliminary rapid analysis. During the debrief, the researchers took note of any new themes emerging to monitor for data saturation and took note of the Ugandan interviewers’ interpretive input on culturally relevant issues which may have emerged. The principal investigator monitored all interviews, reviewing recordings and transcripts for accuracy and quality of interview techniques and providing feedback as necessary on the interview such as probing strategies. Interviewers subsequently transcribed and translated all interviews into English using the audio recording. Notetakers subsequently reviewed and edited transcripts to ensure accuracy. Transcripts were not returned to participants for discussion since participant contact information was not collected to respect confidentiality. No repeat interviews were conducted.

### Data analysis

Two interviewers (JK, SM) and the principal investigator (MBN) worked together to conduct a preliminary rapid analysis of the findings based on the notes for a rapid report of findings with the primary purpose to inform the design of the discrete choice experiment. Interviewers provided insights regarding cultural context as part of the analysis process since they were from Uganda. An adjusted version of an established rapid analysis process was used where the matrices of notes were used to help break down and summarize findings [29]. In this process, matrices were derived from the notes taken from the interviews and input into an excel sheet organized by questions which were grouped into themes. Analysts reviewed the matrices for accuracy utilizing transcripts as needed, summarizing and highlighting important information before creating summaries. Summaries were then utilized to create a report of findings. The report of the rapid analysis findings was made to consult with other Ugandan team members and presentation to the MUWRP CAB for member checking and validation of the results. The rapid analysis summary was also used to form the initial coding tree (S3 File), which was adjusted iteratively during the subsequent thematic analysis of the data.

For the purpose of the thematic analysis, the team used an inductive approach to further develop the coding tree from the rapid analysis and add any new themes that emerged from the data [30]. The rapid analysis was conducted with the purpose of producing a structured systematic summary of candidate attributes, levels, as well as for implementation considerations for the planned DCE and community engagement, requiring a rapid report and preliminary attribute list. Thematic analysis for this manuscript was conducted subsequently and independently on full transcripts, using the rapid analysis coding tree only as an initial scaffold.

The themes and codes were iteratively reviewed throughout the coding process through team discussion as needed. The codebook and transcripts were entered into the qualitative analysis software Dedoose (version 9.0.17) for coding, data management and subsequent analysis (S3 File) [31]. The analysts each independently coded a transcript, which was chosen due to its comprehensiveness of ideas mentioned in the interview. The team then discussed the codebook as they resolved coding discrepancies. All conflicts were resolved through discussion; and when that was not possible, the principal investigator made the final decision regarding the coding.

We present our findings in a narrative analysis using coded data. Findings reported in this manuscript reflect the thematic analysis of transcripts rather than the rapid report. The consolidated criteria for reporting qualitative research (COREQ) checklist was used to help guide the reporting of our findings [32].

### Reflexivity statement

The four interviewers (JK, BN, MS, RM) are Ugandans with experience in qualitative data collection, and one of whom had experience leading qualitative data collection teams and conducting discrete choice experiments (RM). Three of them were men (JK, MS, RM) and one was a woman (BN). Their cultural background was similar to that of participants, although two were from another nearby village. All were fluent in Englis hand Luganda, the primary local language spoken in that region. All researchers had at least a bachelor’s degree in either social work or public health; two were completing master’s in public health at the time of this study (JK, RM), one was working in public service (BN), and two were actively conducting public health research for other projects at the same time (JK, RM). Two were counselors, one was working for the Ministry of Gender, Labour and Social Development, and the other was a project coordinator for a renowned research institute in Uganda. All received additional training on qualitative research before beginning data collection.

The three data analysts who coded the data were all women based at an American institution at the time of the study (MN, WH, CD). One of them was studying for her PhD in public health (MN), one had completed two master’s degrees, one of which was in public health (CD), and the other had completed a Bachelor of Medicine and Surgery (MBBS) and was completing her master’s in public health at the time of data analysis (WH). One of the researchers was from Iran (WH) and the other two were from the USA (MN, CD). Two had prior research knowledge in a global health context from previous research experiences (MN, WH).

The study was approved by the Makerere University School of Public Health Research and Ethics Committee and the Uganda National Council for Science and Technology (SPH-2023–465/HS 3769ES). One protocol deviation took place during this study when photographs were taken of participants with their consent using a consent document that was not approved by the ethical review board to be used for this study. To correct this, the study team deleted all photographs taken during the study.

## Results and discussion

### Participant demographics

Experts were primarily Counselors/ KP Focal Persons (n = 4) but also included one of each of the following positions: HIV Prevention Officer, Team leader, Drop-in Center (DIC) Coordinator, Senior research supervisor, Fieldworker, and Program officer. Experts were selected for their experience supporting HIV prevention programming and key population service delivery in the study districts. Among the expert participants, 50% were female (n = 5), 40% were male (n = 4), and 10% identified as other genders or declined to disclose (n = 1), though specific gender identities were not disclosed.

Among PSRHA participants, peer leaders represented networks of diverse key and priority populations, including men who have sex with men (MSM), adolescent girls and young women (AGYW), transgender men and women, fisherfolk, female sex workers (FSWs), PWIDs, truck drivers, boda-boda drivers, uniformed personnel, and the general population. Of the PSRHA participants, 50% were female (n = 5), 30% were male (n = 3), and 20% identified as other genders (n = 2). PSRHA participants had a mean age of 29.8 years (range 21–42), compared to 40.6 years among experts (range 30–50). Most participants in both groups worked in government settings (PSRHA n = 7; experts n = 8). Participants were primarily based in Kampala (PSRHA n = 7; experts n = 8), with smaller representation from Mukono (PSRHA n = 2; experts n = 1) and Kayunga (PSRHA n = 1; experts n = 1).

In terms of marital status, two PSRHA participants were married, while eight were single or had never been married. Education levels varied, with two PSRHA participants having finished primary school, three having attended some secondary school, and five completing secondary school. Regarding employment status, five PSRHA participants were employed full time, three were employed part time, and two were unemployed. The average number of adults in each participant’s household was 8.6 (median: 3, range: 1–50) while the average number of children was 5.5 (median: 4, range: 0–30). Household counts were highly skewed due to a small number of participants reporting very large households, which is reflected in the higher mean relative to the median.

When asked how many people they provided monetary support for, two PSRHA participants supported one person, three supported two to five people, four supported six to ten people, and one participant supported more than ten people. The average monthly income was 275,000 UGX (~75 USD), with a range from 50,000 (~14 USD) to 700,000 UGX (~190 USD); for context, the international poverty line during this study was approximately 200,000 UGX (~55 USD) [33]. Reported monthly income reflects total household income. In relation to HIV, six participants knew a friend living with HIV, three knew a family member, and one participant did not know anyone living with HIV. Eight participants expressed concern about acquiring HIV, one was somewhat concerned, and one was not concerned at all.

Additionally, eight participants reported having had a sexually transmitted infection (STI) in the past six months, four had taken post-exposure prophylaxis (PEP), six shared injecting materials, and one participant had a partner living with HIV. See demographic information in Table 1.

**Table 1:** Participant demographics for HIV prevention injectable discrete choice experiment key informant interviews, n = 20.

	PSRHA (n = 10) *n (%)*	Experts (n = 10) *n (%)*
Age	Mean: 29.8 (range 21–42)	Mean: 40.6 (range: 30–50)
Gender		
Female	5 (50%)	5 (50%)
Male	3 (30%)	4 (40%)
Other/Refuse	2 (20%)	1 (10%)
Worksite		
Government	7 (70%)	8 (80%)
Nonprofit	2 (20%)	1 (10%)
Clinic	1 (50%)	1 (50%)
Site		
Kampala	7 (70%)	8 (80%)
Mukono	2 (20%)	1 (10%)
Kayunga	1 (50%)	1 (50%)

### Overview of themes

**Fig 1:**
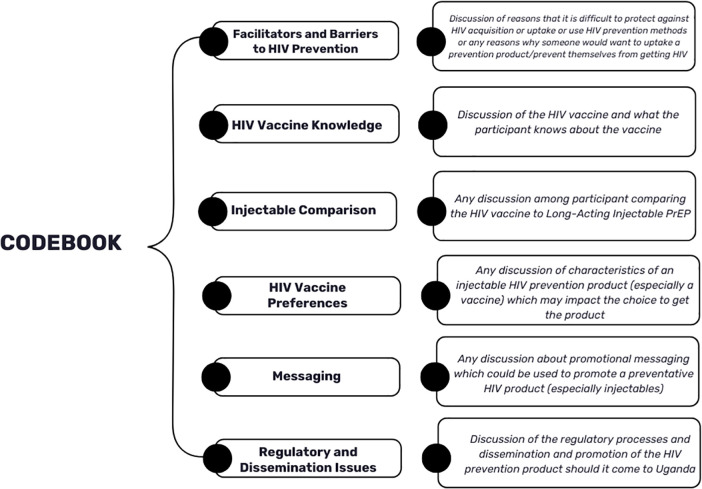
Summary of codes to be discussed in this‌‌ manuscript.

While the codebook for the thematic analysis aligned closely with the guide and its questions, some inductive themes emerged such as some barriers to prevention like the “pill burden”, accessibility issues and stigma-related issues. Topics which were discussed during the interviews fell under six primary themes: Facilitators and Barriers to HIV Prevention, HIV Vaccine Knowledge, Injectable Comparison, HIV Vaccine Preferences, Messaging and Regulatory and Dissemination Issues. It is important to note that the last theme about regulatory and dissemination issues was only discussed by the expert participants. Please see a summary of all codes below in Fig 1. The full codebook is included in S3 File. Findings related to each of these major theme groups are included below.

### Facilitators and barriers to HIV prevention

The most frequently mentioned prevention methods used in communities were condoms and PrEP, followed by post-exposure prophylaxis (PEP) and HIV testing. Several different barriers to HIV prevention were discussed among the participants. These barriers consisted of ‘tablet fatigue’ or ‘pill burden’ availability and stigma for PrEP, and misconceptions, location, accessibility, transportation, and cost for both.

The pill burden barrier coincided frequently with stigma. Stigma as a barrier was discussed in relation to barriers to using certain HIV prevention methods. One concern was the packaging for PrEP being like ART. Many mentioned not wanting to be perceived as taking ART and being misidentified as HIV-infected. One interviewee stated,

*“[P]eople would easily think that you have HIV/AIDS. ‘They think it isn’t for prevention but ART treatment.’” (PKAY09)*

It was mentioned that some key populations put off getting refills of oral PrEP, sometimes due to side effects, and thus use the method on and off. Taking a daily tablet was more difficult for certain key populations, like PWIDs and FSWs, who move around frequently. For those populations that moved around frequently like truck workers, they sometimes forgot to carry their pills with them or were unable to get the medication leading to them missing their daily dose.

The barrier of “tablet fatigue” and “pill burden” coincided frequently with stigma. Stigma as a barrier is discussed in the relation to barriers of using certain HIV prevention methods. One concern is the packaging for PrEP being like ART. Many mentioned not wanting to be perceived as taking ART and being positive. One interviewee stated,

*“[P]eople would easily think that you have HIV/AIDS. ‘They think it isn’t for prevention but ART treatment.’”* (PKAY09)

In a similar vein, one reason people do not adhere to taking the drugs was that they do not want other people to see that they have the pills. Some did not want to disclose to partners, which made it hard for them to take the pills, and not have partners find out. Health centers were discussed as being places where key populations experienced stigmatization:

*“They have their reasons like they will be stigmatized by the health workers and pointing fingers.” (EMUK10)*

For example, it was discussed that transgender people were judged by their physical appearance when visiting the facilities for refills. Some health workers were reported as perceiving young adolescents and young key populations negatively for accessing condoms and PrEP. However, it was mentioned in a few interviews that there were often a few trusted healthcare workers who worked closely with LGBT populations to whom these populations were often delegated. If those trusted healthcare workers were not present, they may not receive treatment that day.

Participants discussed misconceptions regarding different HIV prevention methods. These misconceptions included how these methods affect people’s bodies and the side effects that they were concerned about. One participant, for example, mentioned a misconception among female sex workers that PrEP was brought by the government to get rid of sex workers. Additional misconceptions regarding tablets included that HIV was in the tablets or that the tablets could cause cancer or hallucinations, or affect the liver, fertility, or sexual virility:

*“When we looked at the size of the PrEP tablet, it was just as big as ART pills, so, we thought it was simply an HIV drug, and they were just lying to us, and it was to be administered daily the same way with ART pills.” (PKAY09)*

Location, accessibility and transportation were three barriers that were interconnected in the interviews. Some facilities administering PrEP were mentioned to not be open on the weekends or not have health workers working on the weekends. For those who did not have transportation, picking up refills had become difficult. Long distances also prevented people from accessing the prevention methods they needed. Additionally, in most health facilities, for PrEP to be given, someone must first self-identify as a key population or be at high risk of acquiring HIV, making it difficult for some people to get the PrEP drug, especially those that did not want to disclose their identity due to fear of being stigmatized. This was especially true for LGBT participants. As one participant said,

*“So, there is a barrier for refills, and then there is a barrier of transport for them coming to pick PrEP after every three months or after one month. And then also there is inaccessibility, whereby it is not accessible at every health center within Uganda.” (EKAM02)*

The limited availability of methods was a barrier discussed. Vaginal rings and PrEP are not always available in all health centers. In some instances, key populations were not aware of PrEP availability, this limited information, or even lack of information impacted access to HIV prevention methods.

Lastly, cost was discussed by two participants when it came to prevention methods. In certain hospitals where oral PrEP is sold, it is not affordable. It was mentioned that some do not know where the health facilities are where they can get these items for free. As one participant said,

*“Because any preventive product that is available at a cost sometimes would be avoided as people might not afford to buy them. So, those are the challenges.” (PKAM06)*

Although facilitators were discussed less than barriers, those that were mentioned included that ease and convenience of a method made it more likely that the key populations would utilize the method for prevention of HIV. They also mentioned that a feeling of freedom of fear of either side effects or infection of HIV acquisition was a factor that would incentivize them to take up an HIV prevention product.

### HIV vaccine knowledge

While most participants appeared to have heard about the HIV vaccine, most did not have a lot of specific knowledge about the vaccine. Participants said that the vaccine protected against HIV when having unprotected sex, or would “boost” their body, or their “cells-blood cells” (PMUK10). Others thought the vaccine would act more like antiretroviral medications or a cure, saying it would “stop [HIV] from progressing to AIDS” (EKAY09). Many knew that vaccines were still under clinical trials. A few mentioned that the vaccine could cause side effects:

*“I heard them say that if any vaccine is still new in the body, it first weakens you, it causes dizziness because it is not used to the body. Sometimes you can even get some running whatever.” (PKAM05)*

There was also some confusion regarding the duration of the vaccine, possibly due to remembering information about injectable PrEP; some participants thought the HIV lasted for one to two months, others for six months, others for one year, while others said they did not know. A few seemed to have received information that it *“prevents HIV but it will not prevent other diseases” (*PKAM03) or pregnancy. When asked about mRNA HIV vaccine technology, participants had limited knowledge and understanding of the technology. One expert participant mentioned that they thought everyone would be required to get the vaccine, like the polio vaccine, though others were not sure. Another participant said they thought it would be expensive. One participant additionally mentioned concern that political will may interfere with the delivery of a future HIV vaccine:

*“But if this vaccine is approved and introduced, then I believe everyone would prefer the vaccine instead. So, it would be nice to introduce the vaccine, only those politicians and other opportunists always tend to sabotage such kind of initiatives for their selfish interests.” (PKAM06)*

Participants mentioned a variety of sources from whom they had heard information about the HIV vaccine including peers, friends, health educators, the news, the radio, talks from organizations like USAID, and community dialogues. A few participants mentioned that community talks had tried to preemptively address myths circulating in the community. Misinformation mentioned by participants primarily circulated safety issues (e.g., reduced lifespan, mental problems, reproduction issues). Experts, on the other hand, tended to view these concerns as myths that needed to be addressed through sensitization and education, focusing on the potential of the vaccine to reduce stigma and protect public health.

Participants also mentioned a worry that the HIV vaccine may encourage risky behaviors like unprotected sex, which may increase rates of unplanned pregnancy and STDs as a result:

*“Getting vaccinated against HIV doesn’t mean living more recklessly with life, however, some would do exactly that, left and right, upon realizing that they cannot contract HIV any longer, which is very wrong.” (PKAM06)*

Participants mentioned that key populations generally want the vaccine though, as one expert participant said, *“They welcome it. Ok? They welcome it”* (EKAM05). But there are still worries about safety. A clear message from participants was that key populations need enough information to feel motivated to get the vaccine, especially if that information comes from those doing the research.

### Preferences for injectable PrEP compared to the HIV vaccine

When asked which they would prefer, some preferred a vaccine because they think it will last longer, even for the rest of one’s life “you receive once and for all” (EKAM08). For instance, one participant mentioned,

*“[W]ith vaccination, it would be protection for a longer period of time as opposed to the short while for the preventive injectable PrEP.” (PKAM07)*

An expert acknowledged this duration preference and said that when comparing the two, they thought that people would go for the product that lasts longer. On the other hand, some thought PrEP was preferable because they had been using it and did not know much about the new HIV vaccine. It is also important to note that participants often confused PrEP and the HIV vaccine in conversation. For example, one participant mentioned, *“We are told that PrEP also vaccinates”* (PKAM08).

Participants were asked to talk about different factors that may influence their decision to get an HIV vaccine with prompts about common issues mentioned previously in the literature. A few key findings were that preferences for efficacy were for it to be as high as possible, though minimally acceptable efficacy varied significantly. Still, experts tended to have higher minimal acceptable efficacy percentages than PSRHA participants overall. For example, one participant emphasized willingness to use an option despite low effectiveness: *‘They can use it even 20% we [FSWs] can use it…’* (PKAM08). These divergent thresholds suggest that ‘efficacy’ was not evaluated uniformly; some participants framed it as a non-negotiable requirement, while others prioritized access, discretion, or immediacy of protection. This heterogeneity indicates the need for communication strategies that explain what efficacy percentages mean in practice and for program designs that support informed choice rather than a one-size-fits-all product message.

There was a general preference among participants for the HIV vaccine to be free of cost, though even this preference varied. One participant framed cost as a signal of quality, expressing concern that very low price could be interpreted as low effectiveness, suggesting that free services may still require trusted endorsement and clear messaging about efficacy and safety. It was mentioned that even a small cost for the vaccine would be prohibitive for those unemployed or financially unstable. Some participants mentioned concerns about the safety of the HIV vaccine causing severe health problems. Safety fears included becoming “lame”, getting cancer, liver damage, diabetes, death, infertility, decreased libido, and lower sperm count. Some participants mentioned worries that new vaccines are experimental, perceiving them as a trial conducted on their population. They mentioned a need for proper education and transparency from health workers about the vaccine’s safety and side effects, expressing a desire to see endorsements from health authorities like the Ministry of Health.

As mentioned above, accessibility was a key issue that was identified as a limitation participants anticipated in the rollout of a future HIV vaccine, expressing a preference for lower transportation costs and less documentation requirements, for instance. There was a preference for injections regarding mode of administration due to the perception that they worked faster, were more reliable, and provided long-lasting protection. Concerning the number of doses, many participants favored a single dose that provided lifetime protection to avoid forgetfulness and reduce the number of visits.

Participants preferred taking the HIV vaccine whenever they needed it, emphasizing the importance of ensuring flexibility and convenience. There were varied responses regarding the duration or frequency of doses, but most of the participants preferred quarterly doses, stating that this interval was practical and compatible with existing health practices like family planning injections, while also avoiding multiple frequent visits and site complications.

Regarding the question of who should administer the vaccine, overall, respondents preferred health workers like nurses or doctors to administer vaccines because they were perceived as more trustworthy, knowledgeable, and trained. For the place of administration, many respondents preferred clinics and hospitals because of their accessibility, availability of trained staff, safety, and reliability. Some of the participants preferred community-based locations such as hotspots for the key population like drop-in centers which were seen as safer, and less stigmatizing environments. See a summary of the findings related to preferences in Table 2.

**Table 2:** Summary of expert and PSRHA participant preferences regarding a future HIV vaccine’s characteristics.

Preference	Major findings	Representative Quote
Efficacy	Heterogeneous efficacy thresholds: some articulating a strong ideal for near-perfect protection, while others describing willingness to use a product even with substantially lower effectiveness.	I:, what percentage of effectiveness should it have? R: Effectiveness … 99.9999% and if possible 100%. (PMUK10)
	Possible limited understanding of what effectiveness percentages mean.	“[T]hey can use it even 20% we [FSWs] can use it….” (PKAM08) “R: 50% is an average effectiveness and people would still go for it.” (PKAY09)
Cost	General preference for the HIV vaccine to be free of cost.	“They will not pay.” (EKAM05)
	Contradictory minority opinion that low costs may raise questions about the vaccine’s efficacy.	“If it is cheaper, they will think it is not effective.” (EKAM08)
Safety/Side effects	Concerns about the safety of the HIV vaccine cause severe health problems.	“Yes, you have brought us the vaccine. Won’t it kill us, won’t it make us weak? (PKAM08)
	Concerns about the vaccine causing infertility and sexual health problems.	“So, if I take that vaccine, will I still have that sexual urge?” (EKAM07)
	Worries that new vaccines are experimental.	“They are like whites are doing some trials perhaps on us… We are a dumping what?” (EKAM05)
	Need for proper education and transparency from health workers about the vaccine’s safety and side effects.	“…is it approved by the Ministry of Health?” (EKAM04)
Accessibility/ Availability	Transportation costs and long distances may limit many from getting a future vaccine.	“Now, imagine that kind of person is required to travel some distance for the vaccine and using UGX 20,000 as in transport fare to and from….” (PKAM07)
	Reduction documentation requirements for getting a vaccine and integration into existing healthcare processes will be preferred.	“If there is a lot of documentation that people need to pick from here and there… you do not even mind my name… just give me a vaccine and I go.” (EKAY09)
	Worries about the vaccine running out of stock and potential resulting interruptions of treatment.	“Will it be available whenever I want it? Or it’s just once and it will not be seen again?” (EKAM04)
	Preference for discretion and privacy.	“[P]eople are hiding, and they do not want anything to do with these organizations because they are afraid that they will be arrested or attacked. Yet in the actual sense we are trying to protect them.” (PMUK10)
	Medications should be available for all people, no matter their background or identity.	“…it has to be available for all diversities. Whether for PWDs; people living with disabilities, people who use drugs, availability is very important.” (PMUK10)
Mode of Administration	Preference for injection due to beliefs that injections were more effective than other methods.	“I think they would prefer the injection because in our kind of setting, the perception is that injections work better...” (EKAM06)
	Some preferred drops over injections because of fear of needles or perceptions that they are gentler, less invasive, and less likely to cause side effects.	“They would prefer droplets to injections... [P]eople are more reluctant to get injections. Even if the oral droplets are bitter, it is once in a lifetime people will agree to take them...” (EKAY09)
Site of Administration	Preference for the arm compared to thighs and buttocks due to pain concerns and efficacy perceptions.	“I see the injection on the arm is easier for everybody. Even when people go to the hospital for other illness, they keep saying they want the injection on the arm.” (PKAM08)
Number of Doses	Preference for a single dose that provides lifetime protection to avoid forgetfulness and reduce number of visits.	“Why don’t they make a single dose for life? Because you can forget.” (EKAM03)
On-Demand	Participants preferred taking the HIV vaccine whenever they needed it which will ensure flexibility and convenience.	“I would prefer to have it when I want it, to have it anywhere and easily accessible.” (PKAY09)
	Some thought unrestricted access could lead to misuse or abuse.	“They will misuse it because they will forget they have been affected by substances like marijuana and then request another dose.” (EKAM02)
	A few participants mentioned a scheduled approach for vaccination to ensure compliance.	“If there is a set schedule like one shot every six months, it would ensure compliance.” (EKAM02)
Duration/ Frequency	Preference for quarterly doses to avoid multiple frequent visits and site complications.	“ I would prefer taking it to be every after three months, because after those three months you can come when you no longer have injection pain. And secondly, by the time you go the vaccine will still be in your body because you have not taken long.” (PKAM02)
	Some favored annual/biannual or once in five or ten years because they had busy schedules and wanted less injections and their associated pain and side effects.	“If you give me my injection for 10 years, what challenge do I have with it?” (PKAM08)
	A few preferred a single, lifelong dose to eliminate concerns about frequent visits, and accidentally missing doses.	“I just wish there is a way we can be protected for a lifetime.” (PMUK10)
Person Administering	Preference for health workers like nurses or doctors to administer vaccines.	“Doctors are trusted to keep secrets and provide confidential services.” (PKAM01)
	Some preferred peers and community leaders, but only if trained.	“Peers could be involved if they receive proper training on vaccine administration.” (EKAM06)
Place of Administration	Preference for clinics and hospitals.	“It is better to get the vaccine from the health facility even if it costs, as it ensures safe administration by trained health workers.” (PKAY09)
	Some preferred for community-based locations such as hotspots of the key populations.	“Some prefer receiving services in their hotspots, bars, or places where they feel comfortable, rather than going to health facilities.” (EMUK10)

Across attributes, participants described tradeoffs rather than single-direction preferences. Higher perceived efficacy was often prioritized, but some participants indicated they would accept lower efficacy if the product reduced stigma, required fewer clinic visits, or was easier to access. Similarly, while no-cost access was preferred, some participants expressed concern that very low cost could signal poor quality, indicating that pricing strategies may need to be paired with trusted endorsement and clear messaging.

### Regulatory and dissemination issues

When asked about the regulatory and dissemination processes, some participants responded enthusiastically that a vaccine should be required for all key populations. As one expert participant said,

*“I: Should it be required for all the KPs to take such vaccine for HIV?*

*R: Yes, if its effective and available, yes.” (EKAM07)*

Others were more hesitant about requiring a vaccine but instead emphasized the importance of having a vaccine that is safe and highly effective. Others also said, that instead of requiring a vaccine it could be helpful to have a promotion plan early on to encourage high uptake rates in the at-risk populations. Participants emphasized that people would want to know how a future preventative injection would be disseminated in the future:

*“They would be interested in knowing about the side effects of the vaccine, places of dissemination, availability, and cost. With the condoms, there are really no concerns apart from some brands at some point that they were not comfortable with, that the brand wasn’t good.” (EMUK10)*

Experts mentioned that involving the community will be an important part of the process of getting an HIV vaccine effectively disseminated. They expressed the importance of forming community advisory boards and engaging community leaders to help promote and build confidence among their constituents:

*“So, when we are making policies, we need to involve them, at least we have the community advisory boards, so, if they are involved then to give feedback from the Key Populations themselves, it would help us to get informed on where and how best they would be preferring to get these vaccines.” (EKAM06)*

Several organizations were mentioned that will be key in organizing the process of approval and dissemination including the World Health Organization and other international health organizations will need to approve the product; the institutional review boards, and the Ugandan National Council of Science and Technology, to ensure ethical research is conducted when researching the new products; the National Drug Authority to monitor and approve the drug for distribution in the country; the Ministry of Health to make sure the drug is approved, promoted and disseminated appropriately; pharmacies, health care facilities, drop-in centers, village health teams, peer leaders, and religious leaders to aid in dissemination at a local level. One participant mentioned that some have concerns about receiving preventative products from pharmacies.

### Comparison of expert and PSRHA participant discussions about injectable preferences

**Fig 2:**
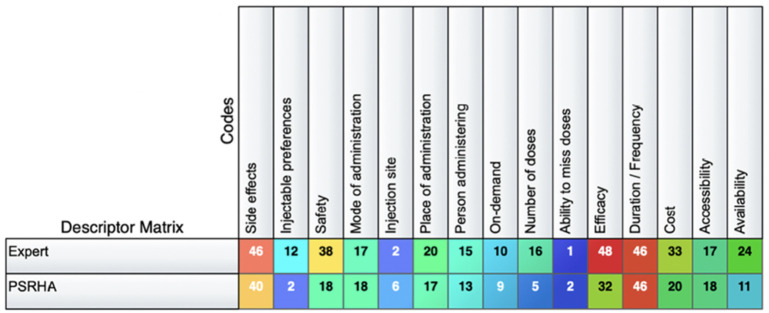
Number of coded segments for expert and PSRHA participants in each group.

Expert and PSRHA participants were asked almost the same questions, however their emphasis in responses were slightly different. For instance, while all participants discussed all preference characteristics, there were more instances of discussions coded about safety, availability, cost and efficacy among experts. On the other hand, there were more coded segments of discussions about the injection site, the ability to miss a dose, the number of doses, and accessibility among PSRHA participants. However, overall, the most discussed preferences among both groups were side effects, duration (i.e., time between doses), efficacy, and cost (Fig 2).

## Discussion

In this study, we found that key populations in Uganda prefer less side effects and consideration of confidentiality in their services. Duration and efficacy were also key attributes which were important in decision making, but preferences varied. A higher efficacy was generally preferred, though the lowest acceptable threshold varied significantly by participant. Although a longer duration was typically preferred, some preferred shorter dosing periods to return more frequently for check-ups and tests. For example, the contradictory finding that shorter dosing periods was preferrable for some participants may be due to the need for more frequent visits and assurance of HIV negativity. This conflicting finding demonstrates the importance of tailored approaches to different populations.

In the interviews, it was clear that key and priority populations needed motivational messaging to increase their interest and willingness to engage in HIV prevention services. The study provides some evidence that participants may not understand the differences between different injectable options, which may indicate that an HIV vaccine may not necessarily be a preferred option if it does not meet sufficient preferred characteristics in other areas in comparison to existing injectable methods of prevention. Additionally, the results of this study provide evidence to demonstrate that the characteristics of the vaccine are not sufficient for optimum uptake of a preventative HIV injectable product. Barriers such as cost, transportation, distance, and social factors like stigma need to be taken into consideration to ensure key populations utilize this strategy for prevention. Finally, this study provides evidence that while many of the barriers and promoters to preventative HIV injectable options for key populations may be structural and out of their control.

Participants emphasized the need for sufficient information about HIV prevention methods to best improve uptake of these products. This is further demonstrated by the finding that some participants appeared to be confused about the differences between the HIV vaccine and injectable PrEP, possibly due to miscommunication from people giving out information in communities or some information may be lost in translation from English to Luganda which does not have an exact translation for vaccine or injectable PrEP. It may also be due to issues with health literacy and the need for additional promotion of information about these products. The observed conflation of PrEP and vaccination suggests that rollout communication for injectable modalities must clearly distinguish mechanism, duration, and continued need for condoms. This is not simply a knowledge gap but a potential driver of mistrust especially if expectations are violated and risk compensation if people assume broad protection. Messaging should therefore use plain-language analogies, explicitly define what efficacy percentages mean, and be delivered through trusted channels endorsed by the Ministry of Health and key-population-facing organizations. A variety of partnering organizations at different levels (e.g., Ministry of Health, nonprofit organizations, peer leaders) were mentioned as being needed to ensure that an HIV vaccine, or any other novel prevention product, is approved and effectively disseminated in a way that inspires trust in the population.

In summary, these findings emphasize the importance of tailored messaging, and of providing choices between different services to accommodate heterogeneity of preferences and best ensure the uptake of HIV prevention services among key and priority populations. There is also a need to consult key populations themselves about HIV prevention issues to ensure that accurate information is attained about preferences as there are slight differences between what the people think compared to what providers, administrators and other experts in the field of HIV prevention think is important. See the summary of practical programmatic recommendations in **Table 3**.

**Table 3:** Practical programmatic recommendations based on results.

Issue	Recommendation
Messaging	Explain efficacy, duration, and the difference between PrEP and vaccine
Trusted endorsement	Collaborative service delivery: Ministry of Health supporting, key population-friendly clinics with trained staff, and peer educators on the front lines
Delivery	Minimize visits, confidentiality protections, flexible hours
Cost	Provide free/subsidized with messaging about quality/safety
Side effects	Provide counseling with support for management pathways

A similar mixed-method study on HIV prevention products found high interest among adolescent girls and young women in an HIV vaccine (34.7%) followed by oral PrEP (25.7%) and injectable PrEP (24.9%) compared to an implant or vaginal ring [17]. Although Mayanja et al. reported the highest product interest for HIV vaccines (34.7%) among adolescent girls and young women, that estimate does not necessarily indicate a dominant preference and may not generalize to key and priority populations represented in the present study [17]. Our sample focused on peer leaders and prevention experts serving diverse key populations, and participants frequently described limited vaccine knowledge and occasional confusion of vaccines with PrEP in everyday conversation, which may shift how interest is expressed and interpreted. Differences may also arise from the outcome being measured (a quantified product ranking versus qualitative narratives about acceptability and implementation concerns) or how questions were framed. Finally, perceived constraints such as stigma, confidentiality, and access barriers may be more salient in key population contexts, potentially shifting expressed preferences toward options viewed as more discreet or easier to sustain.

While this is one of the few studies concerning preferences about a future HIV vaccine in Uganda, there have been several studies that have assessed willingness to participate in HIV vaccine trials, as Uganda is one of the first locations in Africa to engage in HIV vaccine trials [34]. Previous research in Uganda focusing on willingness to participate in HIV vaccine trials have mostly found a high level of willingness (77–95%) to participate in the HIV trials [35–39]. However, this willingness was found to be curbed by concerns about vaccine safety, blood draws, and time required to participate, and increased by concerns of infidelity of current sexual partners [36,40]. These studies suggest an openness to trial participation in some Ugandan settings, but willingness to enroll in a trial should not be interpreted as equivalent to likely real-world vaccine uptake. Importantly, the barriers reported in trial-willingness studies such as safety concerns, time burden, and procedure-related fears align with mechanisms we observed qualitatively, especially the issue of trust, concerns about side effects and experimentation narratives, and the practical constraints of transportation and documentation, which are directly relevant for implementation planning and for designing realistic DCE attributes.

Similar to our findings that higher effectiveness is a preferred attribute of a future injectable product, a discrete choice experiment on the future uptake of PrEP in different forms, including an injection, among fisherfolk in Uganda similarly found preferences for higher effectiveness, although in this study oral PrEP was preferred compared to an injection [20]. This same study also found a significant preference for discretion of administration among men, although not among women, which was also similar to our study’s findings that confidentiality of administration was important for key populations. Another study conducted among key populations in seven countries around the world, including Uganda, found significant importance given to the route of administration for the acceptability of oral PrEP, although side effects did not appear to impact their acceptance [41]. The study additionally emphasized the importance of efficacious and affordability for the participants to accept and uptake the product [41]. This helps to emphasize the importance of lower cost in a future injection product as found in our study, although our findings provide clear indications of possible negative implications of lower cost being interpreted as lower quality. Additionally, a study in rural Uganda and Kenya among demographically diverse populations found that providing patients with dynamic choice during their study increased prevention practices [42]. This demonstrates that person-centered model incorporating structured choice may be an important tool for increasing prevention product uptake overall, similar to our study’s findings of heterogeneous preferences. If preferences vary, more choices should increase preventative practices.

In our study, among PSRHA, convenience issues were a major barrier to accessing prevention services, namely the pill burden, location, transportation, and side effects. Similar barriers which have arisen in other studies regarding PrEP uptake in Uganda include fear of side effects, inconvenience of taking pills often, weakening of the body due to the medication, aversion to taking drugs when healthy, stigma, perceived efficacy, and partner approval [14,43]. In one qualitative study in Uganda and Kenya, PrEP uptake was motivated by high perceived HIV risk and beliefs that PrEP use supported life goals [14]. Another study in Uganda among community health workers found that counseling and motivational interviewing could significantly improve the uptake of PrEP [44].

A key strength of this qualitative work is that it surfaced mechanisms that are difficult to capture in quantitative preference models alone. For example, participants’ narratives illustrated how stigma processes shape access and adherence, how trust and formal endorsement function as prerequisites for uptake, and how numeric concepts such as efficacy and cost may be interpreted through everyday heuristics like conflating prevention technologies or linking low cost with low quality. These findings directly informed the planned DCE by identifying salient attributes like side effects, cost and efficacy, or by clarifying attribute framing and level ranges that are locally meaningful [28].

A few other studies in Uganda have also emphasized the importance of messaging in promoting HIV prevention efforts, similar to our study. For instance the World Health Organization’s MEASURE Evaluation found that it was effective to increase knowledge on prevention measures like condoms to increase disease prevention [45]. Another study demonstrated the importance of tailoring messaging for HIV prevention promotion due to demographic differences leading to differences in misconceptions and attitudes [46]. A study in Mukono, Uganda found that SMS messages as reminders have the potential to increase PrEP adherence which may be a good platform to explore for sending out informational and motivational messages [47]. Our study adds information about additional modes of promotion to the literature such as utilizing PSA messages via voice mail messages and utilizing moral framing tailored to the population of interest to increase acceptability.

A key strength of this qualitative work is that it deepened understanding of HIV prevention preferences by identifying mechanisms underlying stated choices, insights that are difficult to capture through quantitative preference models alone. For example, participants described how stigma, disclosure concerns, and refill constraints such as transportation shape what prevention options are realistically feasible, and how trust and formal endorsement (especially from the Ministry of Health) function as prerequisites for uptake. Interviews also demonstrated how participants interpret numeric concepts like efficacy and cost through heuristic mechanisms which may provide implementation-relevant context for why particular attributes matter and how tradeoffs are evaluated in real-world settings.

Another strength of the study is the breadth of expertise and diversity of participants represented in the sample. This study was additionally conducted at a key point in time, just before the release of a new long-acting PrEP injection in Uganda, right after the end of another unsuccessful yet well publicized HIV Vaccine trial that reached Phase III trials [48], and during that time Discovery Medicine HIV vaccine trials were being launched. A limitation of the study was that there was some risk of response bias, such as social desirability bias, since participants may have wanted to respond in a manner which they thought the researchers wanted to hear [49]. While interviewers were trained on how to increase the comfort of participants and dispel power imbalances, it is impossible to eliminate, especially since there were two researchers in the room. Additionally, higher knowledge among participants may plausibly reflect local exposure to HIV vaccine research infrastructure, since Uganda has longstanding HIV vaccine trial activity and research hubs in places such as Masaka and Entebbe, including participant result-dissemination activities in Masaka in 2024.

The power dynamics may have also been exacerbated due to the Anti-Homosexuality Act issues as mentioned earlier in the article, leading to some participants possibly being more likely to acquiesce in fear of getting in trouble with the law, especially with PSRHA participants. Some participants may have felt inclined to try to please the interviewers by reporting higher willingness to take the HIV vaccine since they knew that was the focus of the research, so our findings of high acceptability of the vaccine should be considered critically and explored further in future studies which may not have as high interaction with facilitators, like anonymous online surveys. In a similar vein, we additionally recognize that having three male interviewers may have altered participants responses at times especially with female participants. We also recognize that since two of the participants were from a nearby city with a slightly different culture, there is the possibility that this may have biased their responses and ability to understand all cultural nuances from the participants when conducting data collection and transcription. One last limitation was that the data analysts for the final thematic analysis were not from Uganda and therefore were less familiar with common practices there, putting the analysis at risk of missing some cultural contexts. However, to remedy this issue, the local Ugandan interviewers, the local Community Advisory Board and other Ugandan researchers were asked to review the analysis findings to ensure all results presented were culturally validated.

Future studies should focus on better understanding the intersectionality between the identities of key populations. In this study, many participants belonged to multiple key populations which made it challenging to understand which issues were unique to one group, or another, or maybe only to those in both groups. More purposeful sampling methods with a lens and a better understanding of the overlap between different key and priority populations in Uganda may help address this issue. Additionally, this study has shown that even before the release of a new product, especially due to the presence of clinical trials in these at-risk communities, people are already talking about new products, setting the groundwork for misinformation to emerge. Therefore, HIV prevention promotion programs should already start ensuring that knowledge about the progress in clinical trials are reported to communities in advance to inoculate against misinformation and ensure that rumors do not spread that are false. Future studies may also explore the added benefits of pairing evidence-based motivations with counseling and motivational interviewing techniques.

Future promotion campaigns for HIV prevention injections should consider revising the messaging strategies to differentiate between PrEP and the HIV vaccine and to promote injectable products focusing on important preferred attributes like effectiveness, side effects and cost which may significantly alter their decision. This could involve using simple, accessible language, avoiding technical jargon, and providing community-specific educational materials that explain how each intervention works, their purpose, and their differences. Messaging should emphasize that if an intervention is free or low cost, it does not indicate a lower quality possibly by transparently revealing the cost and where the funds are coming from to reduce the cost to the target population. Additionally, it is clear the there is a need for educational messaging about how testing for efficacy is conducted to ensure trust in the intervention and possibly providing an option for more frequent checkup visits as needed to provide additional HIV counseling or possibly other services such as primary care or comorbidity (e.g., noncommunicable disease) prevention. Finally, health promotion campaigns should be transparent about the political approval and dissemination process to help build trust and ensure the population knows when and how the product will reach them.

## Supporting information

S1 File Eligibility criteria for key informants. (DOCX)

S2 File Qualitative Interview guide for in-depth interviews with experts. (DOCX)

S3 File Codebook for the HIV prevention injectable discrete choice experiment key informant interviews. (DOCX)
