# Supplementary material for: Exploring HIV prevention preferences among key populations in Uganda: A qualitative study
Source: PLoS One. 2026 Jun 8;21(6):e0349414. doi: 10.1371/journal.pone.0349414 (PMC13245774; doi:10.1371/journal.pone.0349414)
Supplement: S1 File — (DOCX) [file pone.0349414.s001.docx]

**Supplemental File 1 Eligibility criteria for Key Informants**

Participants for the **Expert interview** will be determined eligible for this study if they:

1. Are over the age of 18 years

2. Are able and willing to provide informed consent

3. Are willing to be audio recorded

4. Are available for an appointment to conduct the interview or DCE survey

5. Understand English or Luganda as approved by the IRB

6. Work with people who are considered to be at substantial risk for HIV Acquisition based on the following self-reported characteristics and behaviors

1. Been sexually active in the past six months AND at least one of the following

o you had anal sex in the past six months

o use condoms consistently during sex in the last six months

o had sex with more than one sex partner in the last six months

o had a sex partner in the past six months who is…

§ Living with HIV?

§ A PWID (Person who injects drugs)?

§ A Boda-boda?

§ A Refugee?

§ in prison and closed settings?

§ A recurrent PEP (Post-exposure prophylaxis) user?

§ A Fisher folk?

§ A Long-distance truck driver?

§ A member of Uniformed Forces?

§ An AGYW (Adolescent Girls and Young Women)?

§ A person who has sex with multiple partners without condoms?

§ A PWD (Persons with disabilities in the context of HIV transmission?)

2. If an injection Drug User, they report a history of sharing injection materials/equipment in the past six months.

3. If he/she has had a sexual partner who is HIV+ in the past six months and has not been on effective HIV treatment (i.e., has been on ART for less than six months or has inconsistent or unknown adherence).

Participants for the **PSRHA Interviews** will be determined eligible if they:

1. Are over the age of 18 years

2. Are able and willing to provide informed consent

3. Are available for an appointment to conduct the interview or DCE survey

4. Do understand English or Luganda as approved by the IRB

5. Are considered to be at substantial risk for HIV Acquisition based on the following self-reported characteristics and behaviors

1. Been sexually active in the past six months AND at least one of the following

a. you had anal sex in the past six months

b. use condoms consistently during sex in the last six months

c. had sex with more than one sex partner in the last six months

d. had a sex partner in the past six months who is…

o Living with HIV?

o A PWID (Person who injects drugs)?

o A Boda-boda?

o A Refugee?

o in prison and closed settings?

o A recurrent PEP (Post-exposure prophylaxis) user?

o A Fisher folk?

o A Long distance truck driver?

o A member of Uniformed forces?

o An AGYW (Adolescent Girls and Young Women)?

o A person who has sex with multiple partners without condoms?

o A PWD (Persons with disabilities in the context of HIV transmission?)

2. If an injection Drug User, they report a history of sharing injection materials/equipment in the past six months.

3. If he/she has had a sexual partner who is HIV+ in the past six months and has not been on effective HIV treatment (i.e., has been on ART for less than six months or has inconsistent or unknown adherence).
