# Supplementary material for: Exploring HIV prevention preferences among key populations in Uganda: A qualitative study
Source: PLoS One. 2026 Jun 8;21(6):e0349414. doi: 10.1371/journal.pone.0349414 (PMC13245774; doi:10.1371/journal.pone.0349414)
Supplement: S2 File — (DOCX) [file pone.0349414.s002.docx]

**Supplemental File 2.** Qualitative Interview Guide for In-Depth Interviews with Experts

**Qualitative Interview Guide for In-Depth Interviews**

*Instructions for Researchers: Read aloud the following script to the participant:*

Thank you for agreeing to be part of this interview. We will be talking about your perceptions on a future preventative HIV vaccine and the key populations that you serve, specifically most at risk populations. The time we will spend together is approximately 1 hour. We will also discuss what you know about the HIV vaccines in clinical trials currently and what you think will motivate or prevent young people to take up the preventive HIV vaccine once released. Please feel free to ask me anything that is not clear to you regarding what we will be discussing and remember that there is no right or wrong answer.

Required: HIV prevention in your community – How do people prevent HIV in your community?

Optional probe questions if necessary:

- Tell me what you think people in your community usually use for their HIV preventative measures.
- What do they prefer to use and why? What would they like to use if available and why?
- If they use injectable preventative options like PrEP, why do they use them and what might be barriers to using them?

Required: The HIV Vaccine – What do you know about the HIV vaccine?

Optional probe questions if necessary:

- Please tell me about what you know about the preventative HIV vaccine or about how it works.
- What do you know about the HIV vaccines currently in clinical trials?

A few of the HIV vaccines which made it to clinical trials are mRNA vaccines. What have you heard about mRNA vaccines?

- Are any of them talking about the preventive vaccine and what have you heard them say about the vaccine?

Required: HIV Prevention Preferences:

- Based on your experience, what do you think key and priority populations will consider when deciding whether or not to get the HIV vaccine or other prevention products in the future?

Optional probe questions if necessary:

Side effects

- What do you think taking an HIV vaccine will do inside your body when you are HIV negative?
- How do you think using an HIV vaccine may change anything in your life or in your daily life?
- How do you think taking an HIV vaccine will affect your sexual experience?

Duration of taking vaccine (days, weeks, years, lifetime)

- What length of time do you think is reasonable for one to take an HIV vaccine?

Effectiveness

- What percentage of effectiveness should the HIV vaccine have for people to use it?
- How effective do you think an HIV vaccine should be in preventing HIV infections?

Frequency of administration (once a day vs. before sex acts)

- How often would you prefer to take an HIV vaccine yearly? Every 5 years? Every 10 years? If so, why?
- Would you prefer taking an HIV vaccine on-demand? If so, why?
- How many doses do you think are suitable to take? Why do you think so?
- Would you prefer to get an HIV vaccine as an injectable or drops or other? If so, why?

Cost

- Would you be willing to use an HIV vaccine if you were to pay for it?
- How much would you be willing to pay for the vaccine?
- Will you be willing to use an HIV vaccine if it was available for free?

Places of dissemination (pharmacy, clinic, adolescent/youth center, doctor’s office)

- Tell me about where you think you can get an HIV vaccine when you need to use it?
- How easy do you think it would be for you to get an HIV vaccine? Tell me why you think so.
- Where would you prefer to get an HIV vaccine?

Person who dispenses (doctors, nurses, peer counselor, HIV counselor)

- Who would you prefer to give out the HIV vaccine to you and why?

Optional: Other Questions

Other injectable preventative options

- How would you compare the injectable long-acting PrEP to a vaccine? Is one preferable to the other?

Feasibility of recruitment *(only for experts)*

- What issues do you foresee us possibly facing in recruitment and data collection with the populations of interest for this study?

Required: Messaging:

- What do you think would be a good promotion message that will convince people to get an HIV vaccine?
- Why do you think it is a good message?
- Which channel should be used to promote the message? Why?

Optional: Regulatory, Approval and Dissemination processes *(only for experts)*

- If an HIV vaccine were released, what would the regulatory, approval and dissemination processes look like in Uganda?

Required: Do you have any other thing you want to mention related to this interview before we finish for today?

Do you have any questions for us?
