# Supplementary material for: Exploring HIV prevention preferences among key populations in Uganda: A qualitative study
Source: PLoS One. 2026 Jun 8;21(6):e0349414. doi: 10.1371/journal.pone.0349414 (PMC13245774; doi:10.1371/journal.pone.0349414)
Supplement: S3 File — (DOCX) [file pone.0349414.s003.docx]

**Supplemental File 3.** Codebook for the HIV prevention injectable discrete choice experiment key informant interviews

| **Code** | **Subcode** | **Definition** |
| --- | --- | --- |
| **Themes** | | |
| **Introduction** |  | Introductory remarks by the participant explaining their role, history in HIV prevention work, and any other background information they should choose to provide |
| **Facilitators and barriers to prevention** |  | Discussion of reasons that it is difficult to protect against HIV acquisition or uptake or use HIV prevention methods or any reasons why someone would want to uptake a prevention product/prevent themselves from getting HIV |
|  | **HIV prevention** | Any discussion of HIV prevention methods |
|  | **Stigma** | any discussion of stigma, or the negative views of someone who has HIV or is at risk of HIV, as a barrier to preventing HIV acquisition |
|  | **Oral PrEP packaging** | Discussion of PrEP pills packaging being a barrier to HIV prevention |
|  | **Easy/Convenience** | Discussion of it being "easy" in comparison to other methods |
|  | **Pill burden** | Discussion of the difficulty of having to take so many pills |
|  | **Cost** | Discussion of money or financial constraints or cost of something being a barrier to uptake of HIV prevention products |
|  | **“No Fear”** | Discussion of the freedom of fear of HIV acquisition as an incentive for uptake of a HIV prevention product |
| **HIV Vaccine knowledge** |  | Discussion of the HIV vaccine and what the participant knows about the vaccine |
|  | **mRNA vaccine** | Discussion of mRNA technology and what the participant knows or thinks about it |
|  | **Misinformation** | Any discussion of false information or misinformation |
| **Injectable comparison** |  | Discussion of the difference between vaccine and Long-acting PrEP as the participant understands it or any text which may demonstrate their understanding (or lack thereof) between the two |
| **HIV Vaccine preferences** |  | Any discussion of characteristics of an injectable HIV prevention product (especially a vaccine) which may impact the choice to get the product |
|  | **Efficacy** | Efficacy or effectiveness of the vaccine |
|  | **Cost** | Any discussion of cost as a deciding factor in the choice to uptake an HIV vaccine or long-acting PrEP |
|  | **Side effects** | Any discussion of side effects as a deciding factor in the choice to uptake an HIV vaccine or long-acting PrEP |
|  | **Availability** | Any discussion of the availability of an injectable being a deciding factor in the choice to uptake an HIV vaccine or long-acting PrEP |
|  | **Accessibility** | Any discussion of the accessibility of an injectable being a deciding factor in the choice to uptake an HIV vaccine or long-acting PrEP |
|  | **On-demand** | Availability of the prevention product when needed |
|  | **Duration** | Any discussion of the duration that the injectable will be effective as being a deciding factor in the choice to uptake an HIV vaccine or long-acting PrEP |
|  | **Safety** | Any discussion of safety of an injectable product as being a deciding factor of up taking the prevention product |
|  | **Person administering** | Any discussion of the personnel who will administer the injectable product as being a deciding factor of up taking the prevention product |
|  | **Place of administration** | Any discussion of the place of administration of the injectable product as being a deciding factor of up taking the prevention product |
|  | **Number of doses** | Any discussion of the number of doses of the injectable product as being a deciding factor of up taking the prevention product |
|  | **Mode of administration** | Any discussion of the mode of administration of the injectable product as being a deciding factor of up taking the prevention product |
|  | **Ability to miss doses** | Any discussion of the ability to miss doses of the injectable product as being a deciding factor of up taking the prevention product |
|  | **Ranking** | Discussion where the characteristics driving the decision to get the injectable HIV prevention product are ranked in order of importance |
| **Messaging** |  | Any discussion about promotional messaging which could be used to promote a preventative HIV product (especially injectables) |
|  | **Information** | Discussion of how information about the HIV prevention product is important to include in the message |
|  | **Motivational** | Discussion of how motivational and positive framing of a message about the HIV prevention product is important |
|  | **Suggested messages** | Examples of messages which could be used to promote an HIV prevention product which are suggested by the participant |
|  | **Channels for messaging** | Any discussion of the channels through which HIV prevention product promotional messaging could be sent |
| **Regulatory and Dissemination** |  | Discussion of the regulatory processes and dissemination and promotion of the HIV prevention product should it come to Uganda |
| **Descriptive codes** | | |
| **Key Population: Descriptive code** |  | Discussion of populations that the participant is a part of or works with |
|  | **MSM** | Gay, bisexual and other men who have sex with men |
|  | **AGYW** | Adolescent girls and young women |
|  | **Transgender** | Transgender populations, a person whose current gender does not match their sex assigned at birth |
|  | **Fisher folks** | Fishing communities |
|  | **FSW** | Female sex workers |
|  | **PWID** | People who inject drugs |
|  | **Truck drivers** | long distance truck drivers |
|  | **Boda-bodas** | people who ride on motorcycles to transport people from one place to another |
|  | **Uniformed people** | Any security guards, military personnel, or other professions that wear uniforms |
|  | **General population** | All people in Uganda, since everyone is at risk of HIV acquisition |
| **Injectable: descriptive code** |  | Type of injectable discussed - a descriptive code - can use this code if there is a general discussion about injectables and it is unclear if they are speaking about the HIV vaccine or injectable PrEP |
|  | **HIV vaccine** | Any piece of text discussing the HIV vaccine |
|  | **Long-acting PrEP** | Any piece of text discussing long-acting injectable PrEP |
