# Supplementary material for: Exploring HIV prevention preferences among key populations in Uganda: A qualitative study
Source: PLoS One. 2026 Jun 8;21(6):e0349414. doi: 10.1371/journal.pone.0349414 (PMC13245774; doi:10.1371/journal.pone.0349414)
Supplement: Attachment — Submitted filename: Review the manuscript.pdf [file pone.0349414.s004.pdf]

<u>Reviewer's comments</u>

The abstract does not explicitly define "key populations" (e.g., sex workers, men who have sex with men, people who inject drugs). A brief clarification would enhance clarity. The background could benefit from more emphasis on why understanding preferences for injectables specifically (as opposed to other PrEP forms) is critical in the Ugandan context. The abstract states that participants were "purposively selected" but does not clarify specific criteria for selection (beyond general diversity in groups, genders, occupations, and locations). Were any inclusion/exclusion criteria applied? Although thematic analysis is mentioned, the process is not explained—how themes were identified and how Lancaster's consumer theory was applied remains unclear. While barriers (e.g., stigma, cost, accessibility) are mentioned, specific examples from the data (e.g., illustrative quotes or demographic differences) could strengthen the results section.

Site- The paper states that interviews took place at "MUWRP HIV Clinics and other partnering sites" but does not specify what these partnering sites are or why they were included.

Study population: Were there any restrictions on participant gender, age, or other demographic factors? How were experts selected? Was there a minimum level of experience required? Explain how experts were chosen and whether demographic diversity was considered.

While the Anti-Homosexuality Act is mentioned, it is unclear how these impacted which criteria were excluded from the screening process. Providing a specific example would improve transparency.

The rationale for geographic selection (Kampala, Mukono, Kayunga) is missing—why were these districts chosen over others?

The Community Advisory Board (CAB) is mentioned, but it is unclear what specific role they played in shaping the study design. Were there any adaptations to the study based on their feedback?

While Lancaster's consumer theory is well-described, how exactly was it used in the analysis? The section could clarify how participant responses were mapped to the theory and what specific elements of the Random Utility Model were applied to qualitative findings. Provide examples of how participant responses align with the theory.

Lack of Justification for Data Collection Timeline; Why were interviews conducted within a short timeframe? Explain: If external factors influenced this timeline, and whether this limited timeline affected data saturation or participant recruitment.

While data collection procedures are clear, how were participants recruited?

The authors state that the interview process varied slightly depending on previous interviews and the interviewing style of the four interviewers. This raises concerns about consistency—was there a training protocol to minimize interviewer bias? How were interviewers monitored for adherence to the interview guide?

There is a need for more detail on note-taking and rapid analysis. The matrix format for notes is a strength, but what criteria were used to identify emerging themes? And how were field notes incorporated into the final analysis beyond preliminary insights?

The transcription process lacks detail: Were transcripts verified against audio recordings by an independent reviewer? If not, could errors have been introduced during translation or transcription? The section mentions that transcripts were not returned to participants for verification. While this is understandable due to confidentiality concerns, how was accuracy ensured? The authors do not describe how the data was stored after collection. How was confidentiality maintained post-collection?

A rapid analysis process was used, but why was this chosen over other methods (e.g., framework analysis,)? More detail on how the rapid analysis was adjusted from the original method would improve transparency. The adjustments made to the initial coding tree are not well explained: How were new themes identified and incorporated? How was inter-coder agreement ensured during this iterative process?

Member Checking Process Needs More Detail. How was feedback from the MUWRP CAB incorporated into the analysis? Did any major discrepancies arise between initial findings and CAB feedback? If changes were made, were they systematically tracked? Explain how CAB feedback was integrated into the final analysis.

The Conceptual Mapping Process is Not Well Explained. The section states that findings were mapped using Lancaster's model, but what specific model elements were applied? Was thematic analysis explicitly linked to the model during coding? Clearly show how Lancaster's model was used in analysis.

The Reflexivity statement doesn't address how the researchers' identities might have influenced participant interactions. For example, how might gender dynamics have affected interviews in the Ugandan context? Did having three male interviewers and one female interviewer impact data collection with different participant groups? While you mention that interviewers were Ugandan, there's no reflection on their cultural background relative to study participants (regional, ethnic, or linguistic connections that might have facilitated or hindered data collection). The statement focuses on credentials but doesn't address the researchers' theoretical perspectives or methodological assumptions that might have shaped data collection and analysis.

Result—Lancaster's theory's application appears somewhat disconnected from the findings. The theory is introduced late in the results and doesn't clearly demonstrate how it enhanced the analysis throughout.

The themes are presented sequentially without sufficient integration. How do barriers to HIV prevention relate to preferences for a future vaccine? These connections would strengthen the analysis.

The results primarily report what participants said without sufficient analytical depth about why these preferences exist or what they mean for future vaccine development.

Some concepts like stigma and accessibility are repeated across multiple sections without clear synthesis. The results contain valuable information but would benefit from a more integrated analytical approach that moves beyond description to interpretation and connects findings to broader implications for HIV vaccine development and implementation.

The discussion abandons the Lancaster Consumer Economic Theory framework, this creates a theoretical inconsistency that weakens the paper's conceptual foundation. The discussion could strengthen the paper by explaining how their findings extend, challenge, or refine existing theoretical models of health behavior or consumer choice.

The finding that some participants preferred shorter durations to receive more frequent check-ups deserves deeper analysis, as it contradicts conventional assumptions about convenience preferences.

While the authors note confusion between vaccines and injectable PrEP, they primarily attribute this to translation issues rather than exploring potential implications for health literacy interventions.

There is inadequate analysis of power dynamics. Despite mentioning the Anti-Homosexuality Act in the results, the discussion does not engage with how political and legal contexts might influence vaccine uptake.

Although the authors acknowledge that non-Ugandan data analysts may have missed cultural nuances, they don't sufficiently reflect on how this might have influenced interpretation. The claim that having Ugandan researchers review findings "ensured" cultural validation overstates the effectiveness of this validation approach.

The discussion presents relevant studies but often fails to explicitly connect them to this study's findings, creating a somewhat disjointed narrative. Disproportionate attention is given to literature on PrEP preferences compared to vaccine preferences, the study's primary focus. The flow between different topics (barriers, preferences, messaging) could be improved with clearer transitions and thematic organization.
