# Supplementary material for: Exploring HIV prevention preferences among key populations in Uganda: A qualitative study
Source: PLoS One. 2026 Jun 8;21(6):e0349414. doi: 10.1371/journal.pone.0349414 (PMC13245774; doi:10.1371/journal.pone.0349414)
Supplement: Attachment — Submitted filename: HIV DCE KII Manuscript Reviewer Responses_20250512.docx [file pone.0349414.s006.docx]

Maiya G Block Ngaybe
1914 ½ E 1st St
Tucson, AZ 85719
mgblock@arizona.edu
Phone: +1-520-841-0829

May 12, 2025

Editor-in-Chief
*PLOS One*

Dr. Edward Nicol and reviewers,

## We are writing to thank you and the reviewers for the additional feedback regarding our manuscript titled “*Exploring HIV Prevention Preferences among Key Populations in Uganda: A Qualitative Study.*”

Below, we have provided a table that summarizes each of the reviewers' comments along with our corresponding responses and the changes made to the manuscript.

We once again appreciate the opportunity to improve our work. We hope that the revised manuscript meets the standards of PLOS ONE and look forward to your feedback.

Please do not hesitate to contact us if any further information or clarification is needed. We thank you for considering our submission and are grateful for the time and effort you have invested in our manuscript.

Sincerely,

Maiya G Block Ngaybe

| **Comment** | **Response** |
| --- | --- |
| 1. Please include a complete copy of PLOS’ questionnaire on inclusivity in global research in your revised manuscript. Our policy for research in this area aims to improve transparency in the reporting of research performed outside of researchers’ own country or community. The policy applies to researchers who have travelled to a different country to conduct research, research with Indigenous populations or their lands, and research on cultural artefacts. The questionnaire can also be requested at the journal’s discretion for any other submissions, even if these conditions are not met. Please find more information on the policy and a link to download a blank copy of the questionnaire here: [https://journals.plos.org/plosone/s/best-practices-in-research-reporting](https://url.usb.m.mimecastprotect.com/s/MGB-C2GljPcKm1PE9cMuKf5FI9x?domain=journals.plos.org). Please upload a completed version of your questionnaire as Supporting Information when you resubmit your manuscript. | Completed the questionnaire and made minor edits to the manuscript to comply with the questionnaire. |
| 2. Please note that funding information should not appear in any section or other areas of your manuscript. We will only publish funding information present in the Funding Statement section of the online submission form. Please remove any funding-related text from the manuscript. | The funding section has been moved from the manuscript. |
| 3. Please upload a Response to Reviewers letter which should include a point by point response to each of the points made by the Editor and / or Reviewers. (This should be uploaded as a 'Response to Reviewers' file type.) Please follow this link for more information: [http://blogs.PLOS.org/everyone/2011/05/10/how-to-submit-your-revised-manuscript/](https://url.usb.m.mimecastprotect.com/s/mygmC3Ymkgf74q6REuYCjfQFIla?domain=blogs.plos.org) | Uploading the previous letter with this one under the Response to Reviewers. |
