# Supplementary material for: Exploring HIV prevention preferences among key populations in Uganda: A qualitative study
Source: PLoS One. 2026 Jun 8;21(6):e0349414. doi: 10.1371/journal.pone.0349414 (PMC13245774; doi:10.1371/journal.pone.0349414)
Supplement: Attachment — Submitted filename: KII Cover Letter_20260413.docx [file pone.0349414.s008.docx]

April 13, 2026

Dear Dr. Doherty,

Thank you for the opportunity to submit a revised version of our manuscript, **PONE-D-25-08351R2**, titled **“Exploring HIV prevention preferences among key populations in Uganda: a qualitative study.”** We appreciate your and the reviewer’s careful assessment and constructive feedback. We have revised the manuscript to address all comments, with particular attention to strengthening the contextualization of our findings and clarifying wording and presentation throughout.

Below, we provide a point-by-point response to Reviewer #3. For transparency, we indicate how and where changes were made in the revised manuscript and have ensured that all revisions are reflected in both the tracked-changes version and the clean version.

We also conducted a full manuscript review to ensure consistency between the revised text, tables, and figures, and to address minor grammar/clarity issues throughout. We confirm that we are submitting:

1. a detailed **Response to Reviewers** letter (this document),
2. a **tracked-changes** version of the revised manuscript, and
3. a **clean** version of the revised manuscript.

No changes to the financial disclosure are requested at this time.

Thank you again for your time and consideration. We hope the revisions satisfactorily address all concerns and strengthen the manuscript’s clarity and contextual framing. Please let us know if any additional changes would be helpful.

Sincerely,
Maiya Grace Block Ngaybe, PhD, MPH
(On behalf of all authors)

**Response to Reviewer #3**

**General/contextualization comment**

Abstract:
• In line 47 and 48, it is not clear what “duration” and “location issues” are. Please be more specific. **– tried to clarify more in the text lines 47 and 48.**
• In line 50 and 51, please write this in a simpler way: “Experts tended to have higher minimal acceptable efficacy percentages.” – **Tried to make the wording clearer – lines 50-51**
Introduction:
• In line 77: Rewrite this sentence as follows: “Despite advancements in treatment, HIV remains a major global public health challenge.” – **Reworded as recommended.**
• In line 84: rather say “hindered” instead of “stymied”. It is more formal. – **Reworded as recommended.**
• In line 86: Please also mention the vaginal dapivirene ring as another HIV prevention method even though it has a lower efficacy – **Added this in as recommended.**
• In line 122, remove the word “possible”. – **Removed the word as recommended.**
• In line 132, you have used the acronym DCE higher up in the section so rather write it out in full the first time you use it. – **Reworded as recommended.**

Methods:
• Line 326: Rather specify the country instead of saying “Asia” as you have specified the country for the other two researchers i.e. USA. – **Reworded as recommended.**

Results:
• In Table 1, please specify that you are reporting frequencies. Mean looks like the subheading, but it is actually just for the Age row so it is confusing. I would also add percentages. **-Noted, added additional detail as suggested to the table.**
• You have a lot of text under demographics but very little information in the Table 1. Is it possible to reduce the text and add more of the information in the table? – **Yes, have added more detail.**
• The text in Figure 1 is quite blurry. Is it possible to make it clearer? You could try using AI to assist with the image.  **– I have attached a clearer image**
• In line 398, you mention condoms and PrEP as the most commonly used HIV prevention methods, but most of that section refers to PrEP. Please can you make it clearer, what parts of the results relate to condoms, what part relates to PrEP and which parts relate to both. This could just be in the way you introduce a paragraph. – **Added more clarification.**
• In line 535, please clarify that you are talking about injectable PrEP. Perhaps use the subheading: “Injectable PrEP versus the HIV vaccine” or “Preferences for injectable PrEP compared to the HIV vaccine”. – **Thank you, updated accordingly**
• There are issues with the formatting of Table 2. “Major findings” needs to move over to the left. **– Thank you, updated accordingly.**

Discussion:
• In line 659, again “duration” is unclear as a barrier. Please be more specific. **– added more detail**
• In line 665, it is unusual to put “for example” at the end of a sentence. Please move it to the beginning of the sentence. – **Adjusted accordingly.**
