# Supplementary material for: Exploring HIV prevention preferences among key populations in Uganda: A qualitative study
Source: PLoS One. 2026 Jun 8;21(6):e0349414. doi: 10.1371/journal.pone.0349414 (PMC13245774; doi:10.1371/journal.pone.0349414)
Supplement: Peer review history

# Peer review: Decision Letter 0

**DOI:** 10.1371/journal.pone.0349414.r001

**Authors:** Edward Nicol

24 Mar 2025

PONE-D-25-08351

Exploring HIV prevention preferences among key populations in Uganda: a qualitative study

PLOS ONE

Dear Dr. Block Ngaybe,

Thank you for submitting your manuscript to PLOS ONE. After careful consideration, we feel that it has merit but does not fully meet PLOS ONE’s publication criteria as it currently stands. Therefore, we invite you to submit a revised version of the manuscript that addresses the points raised during the review process.

Your manuscript was reviewed by two expert reviewers, who have provided valuable feedback outlining both its strengths and areas for improvement. They have identified several aspects that require your careful attention and would benefit from revisions. After reviewing your paper myself, I concur with their assessments. Therefore, we invite you to submit a revised version of your manuscript that addresses the points raised during the review process. Please take the time to carefully consider the reviewers' comments as you make your revisions.

Please submit your revised manuscript by May 08 2025 11:59PM. If you will need more time than this to complete your revisions, please reply to this message or contact the journal office at plosone@plos.org. When you're ready to submit your revision, log on to https://www.editorialmanager.com/pone/ and select the 'Submissions Needing Revision' folder to locate your manuscript file.

Please include the following items when submitting your revised manuscript:

- A rebuttal letter that responds to each point raised by the academic editor and reviewer(s). You should upload this letter as a separate file labeled 'Response to Reviewers'.
- A marked-up copy of your manuscript that highlights changes made to the original version. You should upload this as a separate file labeled 'Revised Manuscript with Track Changes'.
- An unmarked version of your revised paper without tracked changes. You should upload this as a separate file labeled 'Manuscript'.

If you would like to make changes to your financial disclosure, please include your updated statement in your cover letter. Guidelines for resubmitting your figure files are available below the reviewer comments at the end of this letter.

If applicable, we recommend that you deposit your laboratory protocols in protocols.io to enhance the reproducibility of your results. Protocols.io assigns your protocol its own identifier (DOI) so that it can be cited independently in the future. For instructions see: https://journals.plos.org/plosone/s/submission-guidelines#loc-laboratory-protocols. Additionally, PLOS ONE offers an option for publishing peer-reviewed Lab Protocol articles, which describe protocols hosted on protocols.io. Read more information on sharing protocols at https://plos.org/protocols?utm_medium=editorial-email&utm_source=authorletters&utm_campaign=protocols.

We look forward to receiving your revised manuscript.

Kind regards,

Edward Nicol, PhD

Academic Editor

PLOS ONE

Journal Requirements:

When submitting your revision, we need you to address these additional requirements.

1. Please ensure that your manuscript meets PLOS ONE's style requirements, including those for file naming. The PLOS ONE style templates can be found at

https://journals.plos.org/plosone/s/file?id=wjVg/PLOSOne_formatting_sample_main_body.pdf and

https://journals.plos.org/plosone/s/file?id=ba62/PLOSOne_formatting_sample_title_authors_affiliations.pdf

2**.** Please include a complete copy of PLOS’ questionnaire on inclusivity in global research in your revised manuscript. Our policy for research in this area aims to improve transparency in the reporting of research performed outside of researchers’ own country or community. The policy applies to researchers who have travelled to a different country to conduct research, research with Indigenous populations or their lands, and research on cultural artefacts. The questionnaire can also be requested at the journal’s discretion for any other submissions, even if these conditions are not met.  Please find more information on the policy and a link to download a blank copy of the questionnaire here: https://journals.plos.org/plosone/s/best-practices-in-research-reporting. Please upload a completed version of your questionnaire as Supporting Information when you resubmit your manuscript.

3. Thank you for stating the following financial disclosure:

“This research was funded by the National Institutes of Health, Fogarty International Center/ Global Health Equity Scholars Fellowship, grant number D43TW010540, the University of Arizona Graduate & Professional Council, and the Mel and Enid Zuckerman College of Public Health Dean’s Fund.”

Please state what role the funders took in the study. If the funders had no role, please state: "The funders had no role in study design, data collection and analysis, decision to publish, or preparation of the manuscript."

If this statement is not correct you must amend it as needed.

Please include this amended Role of Funder statement in your cover letter; we will change the online submission form on your behalf.

4. Thank you for stating the following in the Competing Interests section:

“Authors SM and HK work for the Makerere University Walter Reed Program who actively is con-ducting an HIV vaccine trial. PM was Program Director for the Global Health Equity Scholars Program at the time of this project. The authors declare no other conflicts of interest. The funders had no role in the design of the study; in the collection, analyses, or interpretation of data; in the writing of the manuscript; or in the decision to publish the results.”

Please confirm that this does not alter your adherence to all PLOS ONE policies on sharing data and materials, by including the following statement: "This does not alter our adherence to PLOS ONE policies on sharing data and materials.” (as detailed online in our guide for authors http://journals.plos.org/plosone/s/competing-interests). If there are restrictions on sharing of data and/or materials, please state these. Please note that we cannot proceed with consideration of your article until this information has been declared.

Please include your updated Competing Interests statement in your cover letter; we will change the online submission form on your behalf.

5. In the online submission form, you indicated that “Due to the sensitivity of populations included in this study, the data is not publicly available, but a deidentified version of the data may be requested from the corresponding author.”

All PLOS journals now require all data underlying the findings described in their manuscript to be freely available to other researchers, either 1. In a public repository, 2. Within the manuscript itself, or 3. Uploaded as supplementary information.

This policy applies to all data except where public deposition would breach compliance with the protocol approved by your research ethics board. If your data cannot be made publicly available for ethical or legal reasons (e.g., public availability would compromise patient privacy), please explain your reasons on resubmission and your exemption request will be escalated for approval.

6. When completing the data availability statement of the submission form, you indicated that you will make your data available on acceptance. We strongly recommend all authors decide on a data sharing plan before acceptance, as the process can be lengthy and hold up publication timelines. Please note that, though access restrictions are acceptable now, your entire data will need to be made freely accessible if your manuscript is accepted for publication. This policy applies to all data except where public deposition would breach compliance with the protocol approved by your research ethics board. If you are unable to adhere to our open data policy, please kindly revise your statement to explain your reasoning and we will seek the editor's input on an exemption. Please be assured that, once you have provided your new statement, the assessment of your exemption will not hold up the peer review process.

7. Your ethics statement should only appear in the Methods section of your manuscript. If your ethics statement is written in any section besides the Methods, please move it to the Methods section and delete it from any other section. Please ensure that your ethics statement is included in your manuscript, as the ethics statement entered into the online submission form will not be published alongside your manuscript.

8. Please ensure that you refer to Figure 2 in your text as, if accepted, production will need this reference to link the reader to the figure.

9. Please include captions for your Supporting Information files at the end of your manuscript, and update any in-text citations to match accordingly. Please see our Supporting Information guidelines for more information: http://journals.plos.org/plosone/s/supporting-information.

[Note: HTML markup is below. Please do not edit.]

Reviewers' comments:

Reviewer's Responses to Questions

**Comments to the Author**

1. Is the manuscript technically sound, and do the data support the conclusions?

The manuscript must describe a technically sound piece of scientific research with data that supports the conclusions. Experiments must have been conducted rigorously, with appropriate controls, replication, and sample sizes. The conclusions must be drawn appropriately based on the data presented.

Reviewer #1: Partly

Reviewer #2: Yes

**********

2. Has the statistical analysis been performed appropriately and rigorously?

Reviewer #1: N/A

Reviewer #2: N/A

**********

3. Have the authors made all data underlying the findings in their manuscript fully available?

The PLOS Data policy requires authors to make all data underlying the findings described in their manuscript fully available without restriction, with rare exception (please refer to the Data Availability Statement in the manuscript PDF file). The data should be provided as part of the manuscript or its supporting information, or deposited to a public repository. For example, in addition to summary statistics, the data points behind means, medians and variance measures should be available. If there are restrictions on publicly sharing data—e.g. participant privacy or use of data from a third party—those must be specified.

Reviewer #1: No

Reviewer #2: No

**********

4. Is the manuscript presented in an intelligible fashion and written in standard English?

PLOS ONE does not copyedit accepted manuscripts, so the language in submitted articles must be clear, correct, and unambiguous. Any typographical or grammatical errors should be corrected at revision, so please note any specific errors here.

Reviewer #1: No

Reviewer #2: Yes

**********

5. Review Comments to the Author

Please use the space provided to explain your answers to the questions above. You may also include additional comments for the author, including concerns about dual publication, research ethics, or publication ethics. (Please upload your review as an attachment if it exceeds 20,000 characters)

Reviewer #1: The abstract does not explicitly define “key populations” (e.g., sex workers, men who have sex with men, people who inject drugs). A brief clarification would enhance clarity. The background could benefit from more emphasis on why understanding preferences for injectables specifically (as opposed to other PrEP forms) is critical in the Ugandan context. The abstract states that participants were “purposively selected” but does not clarify specific criteria for selection (beyond general diversity in groups, genders, occupations, and locations). Were any inclusion/exclusion criteria applied? Although thematic analysis is mentioned, the process is not explained—how themes were identified and how Lancaster’s consumer theory was applied remains unclear. While barriers (e.g., stigma, cost, accessibility) are mentioned, specific examples from the data (e.g., illustrative quotes or demographic differences) could strengthen the results section.

Site- The paper states that interviews took place at “MUWRP HIV Clinics and other partnering sites” but does not specify what these partnering sites are or why they were included.

Study population: Were there any restrictions on participant gender, age, or other demographic factors? How were experts selected? Was there a minimum level of experience required? Explain how experts were chosen and whether demographic diversity was considered.

While the Anti-Homosexuality Act is mentioned, it is unclear how these impacted which criteria were excluded from the screening process. Providing a specific example would improve transparency.

The rationale for geographic selection (Kampala, Mukono, Kayunga) is missing—why were these districts chosen over others?

The Community Advisory Board (CAB) is mentioned, but it is unclear what specific role they played in shaping the study design. Were there any adaptations to the study based on their feedback?

While Lancaster’s consumer theory is well-described, how exactly was it used in the analysis? The section could clarify how participant responses were mapped to the theory and what specific elements of the Random Utility Model were applied to qualitative findings. Provide examples of how participant responses align with the theory.

Lack of Justification for Data Collection Timeline; Why were interviews conducted within a short timeframe? Explain: If external factors influenced this timeline, and whether this limited timeline affected data saturation or participant recruitment.

While data collection procedures are clear, how were participants recruited?

The authors state that the interview process varied slightly depending on previous interviews and the interviewing style of the four interviewers. This raises concerns about consistency—was there a training protocol to minimize interviewer bias? How were interviewers monitored for adherence to the interview guide?

There is a need for more detail on note-taking and rapid analysis. The matrix format for notes is a strength, but what criteria were used to identify emerging themes? And how were field notes incorporated into the final analysis beyond preliminary insights?

The transcription process lacks detail: Were transcripts verified against audio recordings by an independent reviewer? If not, could errors have been introduced during translation or transcription? The section mentions that transcripts were not returned to participants for verification. While this is understandable due to confidentiality concerns, how was accuracy ensured? The authors do not describe how the data was stored after collection. How was confidentiality maintained post-collection?

A rapid analysis process was used, but why was this chosen over other methods (e.g., framework analysis,)? More detail on how the rapid analysis was adjusted from the original method would improve transparency. The adjustments made to the initial coding tree are not well explained: How were new themes identified and incorporated? How was inter-coder agreement ensured during this iterative process?

Member Checking Process Needs More Detail. How was feedback from the MUWRP CAB incorporated into the analysis? Did any major discrepancies arise between initial findings and CAB feedback? If changes were made, were they systematically tracked? Explain how CAB feedback was integrated into the final analysis.

The Conceptual Mapping Process is Not Well Explained. The section states that findings were mapped using Lancaster’s model, but what specific model elements were applied? Was thematic analysis explicitly linked to the model during coding? Clearly show how Lancaster’s model was used in analysis.

The Reflexivity statement doesn't address how the researchers' identities might have influenced participant interactions. For example, how might gender dynamics have affected interviews in the Ugandan context? Did having three male interviewers and one female interviewer impact data collection with different participant groups? While you mention that interviewers were Ugandan, there's no reflection on their cultural background relative to study participants (regional, ethnic, or linguistic connections that might have facilitated or hindered data collection). The statement focuses on credentials but doesn't address the researchers' theoretical perspectives or methodological assumptions that might have shaped data collection and analysis.

Result—Lancaster's theory's application appears somewhat disconnected from the findings. The theory is introduced late in the results and doesn't clearly demonstrate how it enhanced the analysis throughout.

The themes are presented sequentially without sufficient integration. How do barriers to HIV prevention relate to preferences for a future vaccine? These connections would strengthen the analysis.

The results primarily report what participants said without sufficient analytical depth about why these preferences exist or what they mean for future vaccine development.

Some concepts like stigma and accessibility are repeated across multiple sections without clear synthesis. The results contain valuable information but would benefit from a more integrated analytical approach that moves beyond description to interpretation and connects findings to broader implications for HIV vaccine development and implementation.

The discussion abandons the Lancaster Consumer Economic Theory framework, this creates a theoretical inconsistency that weakens the paper's conceptual foundation. The discussion could strengthen the paper by explaining how their findings extend, challenge, or refine existing theoretical models of health behavior or consumer choice.

The finding that some participants preferred shorter durations to receive more frequent check-ups deserves deeper analysis, as it contradicts conventional assumptions about convenience preferences.

While the authors note confusion between vaccines and injectable PrEP, they primarily attribute this to translation issues rather than exploring potential implications for health literacy interventions.

There is inadequate analysis of power dynamics. Despite mentioning the Anti-Homosexuality Act in the results, the discussion does not engage with how political and legal contexts might influence vaccine uptake.

Although the authors acknowledge that non-Ugandan data analysts may have missed cultural nuances, they don't sufficiently reflect on how this might have influenced interpretation. The claim that having Ugandan researchers review findings "ensured" cultural validation overstates the effectiveness of this validation approach.

The discussion presents relevant studies but often fails to explicitly connect them to this study's findings, creating a somewhat disjointed narrative. Disproportionate attention is given to literature on PrEP preferences compared to vaccine preferences, the study's primary focus. The flow between different topics (barriers, preferences, messaging) could be improved with clearer transitions and thematic organization.

Reviewer #2: Title: Exploring HIV prevention preferences among key populations in Uganda: a qualitative study.

General comments:

The aim in the abstract (We present data on product preferences for HIV prevention practices and injectable HIV prevention among populations most at risk for HIV with a focus on injectable options such as long-acting PrEP or a future HIV Vaccine for primary prevention of HIV) and in the main body of the manuscript (This study examined factors that are most preferable to at-risk populations, and therefore affect their decisions to accept a preventative HIV vaccine in comparison to other preventative methods with the primary objective to inform the implementation of a discrete choice experiment (DCE) in Uganda) appear to be somehow diffferent.

According to the title the key study participants were key populations. But the study results are presented in such a way that the key populations were taken at the same level as experts.

Appendices 1, 2 &3 were no provided.

Whereas PrEP(Oral & Injectable) is available HIV vaccine is in the future and asking questions to key populations participants to compare the two products may have not been clear to them.

Specific comments

Materials and methods:

Site - the specific sites are not provided here. Though they appear in table 1 of the Results.

Data collection - the authors state that the interview guide used had similar questions though for experts additional questions were included. please elaborate.

Reflexivity - I understand relexivity as a continous process of self-reflection of the Researchers in relation to the ongoing study rather than the experinces and qualifications.

Results:

Numbering after "Overview of themes" needs to be revisited. For consistency maintain the same theme titles as those in the "Figure 1 - summary of codes".

"Facilitators and barriers to HIV prevention" is given as a theme yet only results for "Barriers" are presented.

Discussion:

The discussion is affected by the comment made earlier of putting the key populations and experts in at the same level. My view is that the PRSHA would have been taken as the key study participants and intreviewed first and thereafter conduct key informat interviews with experts. The authors can give an explanation.

**********

6. PLOS authors have the option to publish the peer review history of their article (what does this mean?). If published, this will include your full peer review and any attached files.

If you choose “no”, your identity will remain anonymous but your review may still be made public.

**Do you want your identity to be public for this peer review?** For information about this choice, including consent withdrawal, please see our Privacy Policy.

Reviewer #1: No

Reviewer #2: No

**********

[NOTE: If reviewer comments were submitted as an attachment file, they will be attached to this email and accessible via the submission site. Please log into your account, locate the manuscript record, and check for the action link "View Attachments". If this link does not appear, there are no attachment files.]

While revising your submission, please upload your figure files to the Preflight Analysis and Conversion Engine (PACE) digital diagnostic tool, https://pacev2.apexcovantage.com/. PACE helps ensure that figures meet PLOS requirements. To use PACE, you must first register as a user. Registration is free. Then, login and navigate to the UPLOAD tab, where you will find detailed instructions on how to use the tool. If you encounter any issues or have any questions when using PACE, please email PLOS at figures@plos.org. Please note that Supporting Information files do not need this step.

Attachment Submitted filename: Review the manuscript.pdf

# Author comment: Author response to Decision Letter 1

**DOI:** 10.1371/journal.pone.0349414.r002

13 May 2025

May 08, 2025

Editor-in-Chief

PLOS One

Dr. Edward Nicol and reviewers,

We are writing to thank you and the reviewers for your valuable feedback on our manuscript titled “Exploring HIV Prevention Preferences among Key Populations in Uganda: A Qualitative Study” (Manuscript ID: ) which was submitted to your journal. We have carefully considered all the comments and suggestions provided by the reviewers and have made the necessary revisions to our manuscript.

Below, we have provided a detailed table below that summarizes each of the reviewers' comments along with our corresponding responses and the changes made to the manuscript.

We believe that these revisions have significantly strengthened our manuscript. We appreciate the opportunity to improve our work. We hope that the revised manuscript meets the standards of PLOS ONE and look forward to your feedback.

Please do not hesitate to contact us if any further information or clarification is needed. We sincerely thank you for considering our submission and are grateful for the time and effort you and the reviewers have invested in our manuscript.

Thank you for your attention to this matter.

Sincerely,

Maiya G Block Ngaybe

Attachment Submitted filename: HIV DCE KII Manuscript Reviewer Responses_20250512.docx

# Peer review: Decision Letter 1

**DOI:** 10.1371/journal.pone.0349414.r003

**Authors:** Edward Nicol

11 Jun 2025

PONE-D-25-08351R1

Exploring HIV prevention preferences among key populations in Uganda: a qualitative study

PLOS ONE

Dear Dr. Block Ngaybe,

Thank you for submitting your manuscript to PLOS ONE. After careful consideration, we feel that it has merit but does not fully meet PLOS ONE’s publication criteria as it currently stands. Therefore, we invite you to submit a revised version of the manuscript that addresses the points raised during the review process.

Your manuscript has been re-evaluated by two expert reviewers. While we acknowledge that some earlier comments have been addressed, one of the reviewers remains unsatisfied with aspects of the revised submission and has provided further constructive feedback, which I fully support.

We therefore invite you to submit a revised version of your manuscript that thoroughly addresses the reviewers' concerns. Please take the time to carefully consider all comments and suggestions during your revision process. A detailed response letter outlining how each point has been addressed will be appreciated and will facilitate the re-review.

Please submit your revised manuscript by Jul 26 2025 11:59PM. If you will need more time than this to complete your revisions, please reply to this message or contact the journal office at plosone@plos.org. When you're ready to submit your revision, log on to https://www.editorialmanager.com/pone/ and select the 'Submissions Needing Revision' folder to locate your manuscript file.

Please include the following items when submitting your revised manuscript:

- A rebuttal letter that responds to each point raised by the academic editor and reviewer(s). You should upload this letter as a separate file labeled 'Response to Reviewers'.
- A marked-up copy of your manuscript that highlights changes made to the original version. You should upload this as a separate file labeled 'Revised Manuscript with Track Changes'.
- An unmarked version of your revised paper without tracked changes. You should upload this as a separate file labeled 'Manuscript'.

If you would like to make changes to your financial disclosure, please include your updated statement in your cover letter. Guidelines for resubmitting your figure files are available below the reviewer comments at the end of this letter.

If applicable, we recommend that you deposit your laboratory protocols in protocols.io to enhance the reproducibility of your results. Protocols.io assigns your protocol its own identifier (DOI) so that it can be cited independently in the future. For instructions see: https://journals.plos.org/plosone/s/submission-guidelines#loc-laboratory-protocols. Additionally, PLOS ONE offers an option for publishing peer-reviewed Lab Protocol articles, which describe protocols hosted on protocols.io. Read more information on sharing protocols at https://plos.org/protocols?utm_medium=editorial-email&utm_source=authorletters&utm_campaign=protocols.

We look forward to receiving your revised manuscript.

Kind regards,

Edward Nicol, PhD

Academic Editor

PLOS ONE

[Note: HTML markup is below. Please do not edit.]

Reviewers' comments:

Reviewer's Responses to Questions

**Comments to the Author**

1. If the authors have adequately addressed your comments raised in a previous round of review and you feel that this manuscript is now acceptable for publication, you may indicate that here to bypass the “Comments to the Author” section, enter your conflict of interest statement in the “Confidential to Editor” section, and submit your "Accept" recommendation.

Reviewer #1: (No Response)

Reviewer #2: All comments have been addressed

**********

2. Is the manuscript technically sound, and do the data support the conclusions?

The manuscript must describe a technically sound piece of scientific research with data that supports the conclusions. Experiments must have been conducted rigorously, with appropriate controls, replication, and sample sizes. The conclusions must be drawn appropriately based on the data presented.

Reviewer #1: No

Reviewer #2: Yes

**********

3. Has the statistical analysis been performed appropriately and rigorously?

Reviewer #1: N/A

Reviewer #2: N/A

**********

4. Have the authors made all data underlying the findings in their manuscript fully available?

The PLOS Data policy requires authors to make all data underlying the findings described in their manuscript fully available without restriction, with rare exception (please refer to the Data Availability Statement in the manuscript PDF file). The data should be provided as part of the manuscript or its supporting information, or deposited to a public repository. For example, in addition to summary statistics, the data points behind means, medians and variance measures should be available. If there are restrictions on publicly sharing data—e.g. participant privacy or use of data from a third party—those must be specified.

Reviewer #1: No

Reviewer #2: No

**********

5. Is the manuscript presented in an intelligible fashion and written in standard English?

PLOS ONE does not copyedit accepted manuscripts, so the language in submitted articles must be clear, correct, and unambiguous. Any typographical or grammatical errors should be corrected at revision, so please note any specific errors here.

Reviewer #1: Yes

Reviewer #2: Yes

**********

6. Review Comments to the Author

Please use the space provided to explain your answers to the questions above. You may also include additional comments for the author, including concerns about dual publication, research ethics, or publication ethics. (Please upload your review as an attachment if it exceeds 20,000 characters)

Reviewer #1: The study addresses a timely and important topic - understanding preferences for injectable HIV prevention methods in Uganda before their rollout. The mixed-methods approach, incorporating both expert and community perspectives, provides valuable triangulation of viewpoints.

The reference to "Lancaster's consumer theory" as the analytical framework feels somewhat misaligned with the qualitative nature of the study. Consumer choice theory typically applies to quantitative preference modeling, and it's unclear how this guided the thematic analysis described.

The introduction feels somewhat disjointed, as it jumps between different topics without smooth transitions. The paragraph structure could be improved; for instance, the discussion of prevention technologies is awkwardly split, and the discussion of vaccine development disrupts the flow about injectable PrEP.

The introduction wavers between discussing HIV prevention broadly and focusing specifically on injectable options. The vaccine discussion appears to be divergent, as the study seems to focus on currently available technologies. There is no mention of Uganda's specific HIV prevention landscape or current PrEP availability. There is limited discussion on why injectable options might be particularly relevant in the Ugandan context. No reference to existing preference research or discrete choice experiments in similar settings

The referencing is inconsistent, with some citations using brackets [1,2] while others use parentheses (6). Citation [8] appears to be used incorrectly, as it's cited for claims that seem to be general statements rather than specific research findings.

Methods - The most significant issue is the use of Lancaster's Consumer Theory in a qualitative study. This economic theory is intended for quantitative preference modeling and discrete choice experiments, not for thematic analysis of interview data. The authors acknowledge that their work is intended to inform a future DCE; however, applying Lancaster's theory to analyze qualitative interviews is methodologically inappropriate and leads to conceptual confusion throughout. The authors should utilize established qualitative frameworks rather than imposing Lancaster's theory on the interview data. Better explain how this qualitative work relates to the planned DCE.

The citation supporting the sample size (reference 12) seems to be misapplied. The authors claim that 16 interviews identify "common themes," while 20 identify "meta themes"; however, this distinction is unclear and may not accurately reflect the findings stated in the cited literature. The rationale for the sample size appears post-hoc rather than theoretically grounded.

The "rapid analysis" process is insufficiently described. The relationship between the rapid analysis for the DCE and the thematic analysis presented in this manuscript remains unclear, which may lead to analytical confusion. There is no discussion of factors related to variations in interview duration, specific probing strategies, or how the cultural context was systematically integrated into the analysis, aside from the use of Ugandan interviewers.

Results - As previously mentioned, the most significant issue is the forced application of Lancaster's Consumer Theory in Section 3.7. This economic theory is fundamentally incompatible with qualitative thematic analysis. The authors attempt to fit their qualitative findings into an economic utility model, which results in conceptual confusion and undermines the value of their qualitative data. The "error term" explanation for social and contextual factors is particularly problematic - these factors are central findings.

Section numbering is confusing (3.6 appears twice), and the flow between sections feels disjointed. There is substantial overlap between sections, particularly around preferences and barriers. The distinction between sections 3.5 and 3.6 is unclear. Key demographic details are absent, such as which specific key populations the PSRHA participants represent, and the table mentions information not discussed in the text.

While many themes are identified, the analysis remains largely descriptive. There is limited exploration of how themes interconnect or their deeper implications for HIV prevention programming. Many quotes feel isolated from a broader analytical context. Some quotes are unclear or do not strongly support the points being made. E.g., the text states there's a "preference for 99.9% or 100% efficacy" but then provides this quote:

"They can use it even 20% we [FSWs] can use it…." (PKAM08)

This quote contradicts the stated finding. It demonstrates a willingness to accept very low efficacy rather than a preference for near-perfect efficacy. The authors do not clarify this contradiction or examine what it means that some participants would accept 20% while others want 100%.

Under cost preferences, they state people prefer free vaccines, then include:

"If it is cheaper, they will think it is not effective." (EKAM08)

This quote suggests a complex relationship between cost and perceived quality, yet the authors do not examine this tension. Could this imply that free vaccines might be turned down? This issue warrants deeper exploration instead of merely listing it alongside "preference for free."

The discussion essentially restates findings without thoroughly exploring their implications. For example, the confusion between PrEP and vaccines is mentioned but not analyzed in terms of its significance regarding health literacy, communication strategies, or intervention design. It doesn't address key contradictions in the data, such as participants wanting both 100% and accepting 20% efficacy, or preferring free products while worrying that cheap products are ineffective.

The literature cited primarily focuses on supportive findings while overlooking contradictory evidence. The comparison with the mixed-methods study, which shows 34.7% interest in HIV vaccines, lacks a critical analysis of why this differs from their findings. Although literature is cited, it is not meaningfully integrated. For example, citing willingness to participate in trials (77-95%) does not necessarily translate to real-world vaccine uptake preferences.

Although limitations are noted, there is a lack of reflection on how the qualitative methodology deepened the understanding of preferences compared to quantitative approaches, as well as what unique insights arose from this method.

Reviewer #2: I have read through the revised manuscript. The Authors have addressed the comments I raised concerning the first manuscript.

**********

7. PLOS authors have the option to publish the peer review history of their article (what does this mean?). If published, this will include your full peer review and any attached files.

If you choose “no”, your identity will remain anonymous but your review may still be made public.

**Do you want your identity to be public for this peer review?** For information about this choice, including consent withdrawal, please see our Privacy Policy.

Reviewer #1: No

Reviewer #2: No

**********

[NOTE: If reviewer comments were submitted as an attachment file, they will be attached to this email and accessible via the submission site. Please log into your account, locate the manuscript record, and check for the action link "View Attachments". If this link does not appear, there are no attachment files.]

While revising your submission, please upload your figure files to the Preflight Analysis and Conversion Engine (PACE) digital diagnostic tool, https://pacev2.apexcovantage.com/. PACE helps ensure that figures meet PLOS requirements. To use PACE, you must first register as a user. Registration is free. Then, login and navigate to the UPLOAD tab, where you will find detailed instructions on how to use the tool. If you encounter any issues or have any questions when using PACE, please email PLOS at figures@plos.org. Please note that Supporting Information files do not need this step.

# Author comment: Author response to Decision Letter 2

**DOI:** 10.1371/journal.pone.0349414.r004

29 Dec 2025

Maiya G Block Ngaybe

1914 ½ E 1st St

Tucson, AZ 85719

mgblock@arizona.edu

Phone: +1-520-841-0829

December 28, 2025

Editor-in-Chief

PLOS One

Dr. Edward Nicol and reviewers,

We are writing to thank you and the reviewers for the additional feedback regarding our manuscript titled “Exploring HIV Prevention Preferences among Key Populations in Uganda: A Qualitative Study.”

In our cover letter, we have provided a table that summarizes each of the reviewers' comments along with our corresponding responses and the changes made to the manuscript.

We once again appreciate the opportunity to improve our work. We hope that the revised manuscript meets the standards of PLOS ONE and look forward to your feedback.

Please do not hesitate to contact us if any further information or clarification is needed. We thank you for considering our submission and are grateful for the time and effort you have invested in our manuscript.

Sincerely,

Maiya G Block Ngaybe

# Peer review: Decision Letter 2

**DOI:** 10.1371/journal.pone.0349414.r005

**Authors:** Tanya Doherty

17 Feb 2026

PONE-D-25-08351R2

Exploring HIV prevention preferences among key populations in Uganda: a qualitative study

PLOS One

Dear Dr. Block Ngaybe,

Thank you for submitting your manuscript to PLOS ONE. After careful consideration, we feel that it has merit but does not fully meet PLOS ONE’s publication criteria as it currently stands. Therefore, we invite you to submit a revised version of the manuscript that addresses the points raised during the review process.

Please address the points raised by the reviewer especially around contextualising your research.

Please submit your revised manuscript by Apr 03 2026 11:59PM. If you will need more time than this to complete your revisions, please reply to this message or contact the journal office at plosone@plos.org. When you're ready to submit your revision, log on to https://www.editorialmanager.com/pone/ and select the 'Submissions Needing Revision' folder to locate your manuscript file.

Please include the following items when submitting your revised manuscript:

- A letter that responds to each point raised by the academic editor and reviewer(s). You should upload this letter as a separate file labeled 'Response to Reviewers'.
- A marked-up copy of your manuscript that highlights changes made to the original version. You should upload this as a separate file labeled 'Revised Manuscript with Track Changes'.
- An unmarked version of your revised paper without tracked changes. You should upload this as a separate file labeled 'Manuscript'.

-->If you would like to make changes to your financial disclosure, please include your updated statement in your cover letter. Guidelines for resubmitting your figure files are available below the reviewer comments at the end of this letter.

If applicable, we recommend that you deposit your laboratory protocols in protocols.io to enhance the reproducibility of your results. Protocols.io assigns your protocol its own identifier (DOI) so that it can be cited independently in the future. For instructions see: https://journals.plos.org/plosone/s/submission-guidelines#loc-laboratory-protocols. Additionally, PLOS ONE offers an option for publishing peer-reviewed Lab Protocol articles, which describe protocols hosted on protocols.io. Read more information on sharing protocols at https://plos.org/protocols?utm_medium=editorial-email&utm_source=authorletters&utm_campaign=protocols.

We look forward to receiving your revised manuscript.

Kind regards,

Tanya Doherty, PhD

Academic Editor

PLOS One

Journal Requirements:

If the reviewer comments include a recommendation to cite specific previously published works, please review and evaluate these publications to determine whether they are relevant and should be cited. There is no requirement to cite these works unless the editor has indicated otherwise.

Please review your reference list to ensure that it is complete and correct. If you have cited papers that have been retracted, please include the rationale for doing so in the manuscript text, or remove these references and replace them with relevant current references. Any changes to the reference list should be mentioned in the rebuttal letter that accompanies your revised manuscript. If you need to cite a retracted article, indicate the article’s retracted status in the References list and also include a citation and full reference for the retraction notice.

[Note: HTML markup is below. Please do not edit.]

Reviewers' comments:

Reviewer's Responses to Questions

**Comments to the Author**

1. If the authors have adequately addressed your comments raised in a previous round of review and you feel that this manuscript is now acceptable for publication, you may indicate that here to bypass the “Comments to the Author” section, enter your conflict of interest statement in the “Confidential to Editor” section, and submit your "Accept" recommendation.

Reviewer #3: (No Response)

**********

2. Is the manuscript technically sound, and do the data support the conclusions?

The manuscript must describe a technically sound piece of scientific research with data that supports the conclusions. Experiments must have been conducted rigorously, with appropriate controls, replication, and sample sizes. The conclusions must be drawn appropriately based on the data presented.

Reviewer #3: Yes

**********

3. Has the statistical analysis been performed appropriately and rigorously?

Reviewer #3: N/A

**********

4. Have the authors made all data underlying the findings in their manuscript fully available?

The PLOS Data policy requires authors to make all data underlying the findings described in their manuscript fully available without restriction, with rare exception (please refer to the Data Availability Statement in the manuscript PDF file). The data should be provided as part of the manuscript or its supporting information, or deposited to a public repository. For example, in addition to summary statistics, the data points behind means, medians and variance measures should be available. If there are restrictions on publicly sharing data—e.g. participant privacy or use of data from a third party—those must be specified.

Reviewer #3: Yes

**********

5. Is the manuscript presented in an intelligible fashion and written in standard English?

PLOS ONE does not copyedit accepted manuscripts, so the language in submitted articles must be clear, correct, and unambiguous. Any typographical or grammatical errors should be corrected at revision, so please note any specific errors here.

Reviewer #3: Yes

**********

6. Review Comments to the Author

Please use the space provided to explain your answers to the questions above. You may also include additional comments for the author, including concerns about dual publication, research ethics, or publication ethics. (Please upload your review as an attachment if it exceeds 20,000 characters)

Reviewer #3: This is an interesting paper and the findings provide important information for those conducting HIV vaccine trials. However, the study seems quite speculative as we still seem quite far from having an approved HIV vaccine and hence not many people would know about the HIV vaccine, unlike injectable PrEP which has been approved for use. It would be good if you could give more context in the introduction on what the status of the HIV vaccine and injectable PrEP are in Uganda and when the public can expect to be able to access them. That being said, it seems that the study site is in an area where HIV vaccine trials are common and so perhaps participants have more knowledge about the HIV vaccine than others and are also likely to be involved in the trials. Please provide this context.

Abstract:

• In line 47 and 48, it is not clear what “duration” and “location issues” are. Please be more specific.

• In line 50 and 51, please write this in a simpler way: “Experts tended to have higher minimal acceptable efficacy percentages.”

Introduction:

• In line 77: Rewrite this sentence as follows: “Despite advancements in treatment, HIV remains a major global public health challenge.”

• In line 84: rather say “hindered” instead of “stymied”. It is more formal.

• In line 86: Please also mention the vaginal dapivirene ring as another HIV prevention method even though it has a lower efficacy

• In line 122, remove the word “possible”.

• In line 132, you have used the acronym DCE higher up in the section so rather write it out in full the first time you use it.

Methods:

• Line 326: Rather specify the country instead of saying “Asia” as you have specified the country for the other two researchers i.e. USA.

Results:

• In Table 1, please specify that you are reporting frequencies. Mean looks like the subheading, but it is actually just for the Age row so it is confusing. I would also add percentages.

• You have a lot of text under demographics but very little information in the Table 1. Is it possible to reduce the text and add more of the information in the table?

• The text in Figure 1 is quite blurry. Is it possible to make it clearer? You could try using AI to assist with the image.

• In line 398, you mention condoms and PrEP as the most commonly used HIV prevention methods, but most of that section refers to PrEP. Please can you make it clearer, what parts of the results relate to condoms, what part relates to PrEP and which parts relate to both. This could just be in the way you introduce a paragraph.

• In line 535, please clarify that you are talking about injectable PrEP. Perhaps use the subheading: “Injectable PrEP versus the HIV vaccine” or “Preferences for injectable PrEP compared to the HIV vaccine”.

• There are issues with the formatting of Table 2. “Major findings” needs to move over to the left.

Discussion:

• In line 659, again “duration” is unclear as a barrier. Please be more specific.

• In line 665, it is unusual to put “for example” at the end of a sentence. Please move it to the beginning of the sentence.

**********

7. PLOS authors have the option to publish the peer review history of their article (what does this mean?). If published, this will include your full peer review and any attached files.

If you choose “no”, your identity will remain anonymous but your review may still be made public.

**Do you want your identity to be public for this peer review?** For information about this choice, including consent withdrawal, please see our Privacy Policy.

Reviewer #3: No

**********

[NOTE: If reviewer comments were submitted as an attachment file, they will be attached to this email and accessible via the submission site. Please log into your account, locate the manuscript record, and check for the action link "View Attachments". If this link does not appear, there are no attachment files.]

To ensure your figures meet our technical requirements, please review our figure guidelines: https://journals.plos.org/plosone/s/figures

You may also use PLOS’s free figure tool, NAAS, to help you prepare publication quality figures: https://journals.plos.org/plosone/s/figures#loc-tools-for-figure-preparation.

NAAS will assess whether your figures meet our technical requirements by comparing each figure against our figure specifications.

# Author comment: Author response to Decision Letter 3

**DOI:** 10.1371/journal.pone.0349414.r006

24 Apr 2026

April 13, 2026

Dear Dr. Doherty,

Thank you for the opportunity to submit a revised version of our manuscript, PONE-D-25-08351R2, titled “Exploring HIV prevention preferences among key populations in Uganda: a qualitative study.” We appreciate your and the reviewer’s careful assessment and constructive feedback. We have revised the manuscript to address all comments, with particular attention to strengthening the contextualization of our findings and clarifying wording and presentation throughout.

Below, we provide a point-by-point response to Reviewer #3. For transparency, we indicate how and where changes were made in the revised manuscript and have ensured that all revisions are reflected in both the tracked-changes version and the clean version.

We also conducted a full manuscript review to ensure consistency between the revised text, tables, and figures, and to address minor grammar/clarity issues throughout. We confirm that we are submitting:

1. a detailed Response to Reviewers letter (this document),

2. a tracked-changes version of the revised manuscript, and

3. a clean version of the revised manuscript.

No changes to the financial disclosure are requested at this time.

Thank you again for your time and consideration. We hope the revisions satisfactorily address all concerns and strengthen the manuscript’s clarity and contextual framing. Please let us know if any additional changes would be helpful.

Sincerely,

Maiya Grace Block Ngaybe, PhD, MPH

(On behalf of all authors)

Response to Reviewer #3

General/contextualization comment

Abstract:

• In line 47 and 48, it is not clear what “duration” and “location issues” are. Please be more specific. – tried to clarify more in the text lines 47 and 48.

• In line 50 and 51, please write this in a simpler way: “Experts tended to have higher minimal acceptable efficacy percentages.” – Tried to make the wording clearer – lines 50-51

Introduction:

• In line 77: Rewrite this sentence as follows: “Despite advancements in treatment, HIV remains a major global public health challenge.” – Reworded as recommended.

• In line 84: rather say “hindered” instead of “stymied”. It is more formal. – Reworded as recommended.

• In line 86: Please also mention the vaginal dapivirene ring as another HIV prevention method even though it has a lower efficacy – Added this in as recommended.

• In line 122, remove the word “possible”. – Removed the word as recommended.

• In line 132, you have used the acronym DCE higher up in the section so rather write it out in full the first time you use it. – Reworded as recommended.

Methods:

• Line 326: Rather specify the country instead of saying “Asia” as you have specified the country for the other two researchers i.e. USA. – Reworded as recommended.

Results:

• In Table 1, please specify that you are reporting frequencies. Mean looks like the subheading, but it is actually just for the Age row so it is confusing. I would also add percentages. -Noted, added additional detail as suggested to the table.

• You have a lot of text under demographics but very little information in the Table 1. Is it possible to reduce the text and add more of the information in the table? – Yes, have added more detail.

• The text in Figure 1 is quite blurry. Is it possible to make it clearer? You could try using AI to assist with the image. – I have attached a clearer image

• In line 398, you mention condoms and PrEP as the most commonly used HIV prevention methods, but most of that section refers to PrEP. Please can you make it clearer, what parts of the results relate to condoms, what part relates to PrEP and which parts relate to both. This could just be in the way you introduce a paragraph. – Added more clarification.

• In line 535, please clarify that you are talking about injectable PrEP. Perhaps use the subheading: “Injectable PrEP versus the HIV vaccine” or “Preferences for injectable PrEP compared to the HIV vaccine”. – Thank you, updated accordingly

• There are issues with the formatting of Table 2. “Major findings” needs to move over to the left. – Thank you, updated accordingly.

Discussion:

• In line 659, again “duration” is unclear as a barrier. Please be more specific. – added more detail

• In line 665, it is unusual to put “for example” at the end of a sentence. Please move it to the beginning of the sentence. – Adjusted accordingly.

Attachment Submitted filename: KII Cover Letter_20260413.docx

# Editor report: Decision Letter 3

**DOI:** 10.1371/journal.pone.0349414.r007

**Authors:** Tanya Doherty

30 Apr 2026

Exploring HIV prevention preferences among key populations in Uganda: a qualitative study

PONE-D-25-08351R3

Dear Dr. Block Ngaybe,

We’re pleased to inform you that your manuscript has been judged scientifically suitable for publication and will be formally accepted for publication once it meets all outstanding technical requirements.

Within one week, you’ll receive an e-mail detailing the required amendments. When these have been addressed, you’ll receive a formal acceptance letter and your manuscript will be scheduled for publication.

An invoice will be generated when your article is formally accepted. Please note, if your institution has a publishing partnership with PLOS and your article meets the relevant criteria, all or part of your publication costs will be covered. Please make sure your user information is up-to-date by logging into Editorial Manager at Editorial Manager® and clicking the ‘Update My Information' link at the top of the page. For questions related to billing, please contact billing support.

If your institution or institutions have a press office, please notify them about your upcoming paper to help maximize its impact. If they’ll be preparing press materials, please inform our press team as soon as possible -- no later than 48 hours after receiving the formal acceptance. Your manuscript will remain under strict press embargo until 2 pm Eastern Time on the date of publication. For more information, please contact onepress@plos.org.

Kind regards,

Tanya Doherty, PhD

Academic Editor

PLOS One

Additional Editor Comments (optional):

Reviewers' comments:

# Editor report: Acceptance letter

**DOI:** 10.1371/journal.pone.0349414.r008

**Authors:** Tanya Doherty

PONE-D-25-08351R3

PLOS One

Dear Dr. Block Ngaybe,

I'm pleased to inform you that your manuscript has been deemed suitable for publication in PLOS One. Congratulations! Your manuscript is now being handed over to our production team.

At this stage, our production department will prepare your paper for publication. This includes ensuring the following:

* All references, tables, and figures are properly cited

* All relevant supporting information is included in the manuscript submission,

* There are no issues that prevent the paper from being properly typeset

You will receive further instructions from the production team, including instructions on how to review your proof when it is ready. Please keep in mind that we are working through a large volume of accepted articles, so please give us a few days to review your paper and let you know the next and final steps.

Lastly, if your institution or institutions have a press office, please let them know about your upcoming paper now to help maximize its impact. If they'll be preparing press materials, please inform our press team within the next 48 hours. Your manuscript will remain under strict press embargo until 2 pm Eastern Time on the date of publication. For more information, please contact onepress@plos.org.

You will receive an invoice from PLOS for your publication fee after your manuscript has reached the completed accept phase. If you receive an email requesting payment before acceptance or for any other service, this may be a phishing scheme. Learn how to identify phishing emails and protect your accounts at https://explore.plos.org/phishing.

If we can help with anything else, please email us at customercare@plos.org.

Thank you for submitting your work to PLOS ONE and supporting open access.

Kind regards,

PLOS ONE Editorial Office Staff

on behalf of

Professor Tanya Doherty

Academic Editor

PLOS One
